# Ultrabright and narrowband organic afterglow achieved by molecular engineering of coronene

**DOI:** 10.1039/d5sc08966b

**Published:** 2026-01-09

**Authors:** Yuanyuan Chen, Yue Zhang, Guoyi Wu, Ting Luo, Jialiang Jiang, Tengyue Wang, Xiaoya Guo, Kaka Zhang

**Affiliations:** a Department of Chemical Engineering, Shanghai University Shanghai 200444 People's Republic of China gxy@shu.edu.cn; b State Key Laboratory of Organometallic Chemistry and Shanghai-Hong Kong Joint Laboratory in Chemical Synthesis, Key Laboratory of Synthetic and Self-Assembly Chemistry for Organic Functional Molecules, Ningbo Zhongke Creation Center of New Materials, Shanghai Institute of Organic Chemistry, University of Chinese Academy of Sciences, Chinese Academy of Sciences 345 Lingling Road Shanghai 200032 People's Republic of China zhangkaka@sioc.ac.cn

## Abstract

Achieving simultaneously ultrabright and narrowband organic afterglow emission remains a formidable challenge because enhancing one property often compromises the other. Herein, we report a low-frequency vibronic coupling strategy to design intrinsic narrowband organic afterglow materials that combine high brightness with long lifetimes. Through molecular engineering of coronene, introduction of aroyl and ethoxy substituents breaks molecular symmetry, enhances intersystem crossing, and preserves a localized triplet state with a small radiative rate constant. The optimized coronene derivative doped into dimethyl isophthalate (DMI) exhibits a phosphorescence efficiency of 17.5%, a lifetime of 3.19 s, and a narrow full width at half maximum (FWHM) of 24.2 nm under ambient conditions. Deuteration further increases the lifetime to 5.11 s and phosphorescence efficiency to 20.2%, achieving one of the brightest (73 cd m^−2^ at ∼0.2 s) and narrowband organic afterglow emissions to date. Theoretical simulations attribute the narrowband emission to dominant low-frequency vibronic coupling in the coronene derivative system, offering mechanistic insights into spectral narrowing. This work establishes a new paradigm for molecular design of ultrabright narrowband afterglow materials, paving the way for their applications in illumination, anticounterfeiting, and information encryption.

## Abstract 2

Ultrabright and narrowband organic afterglow is achieved using engineered coronene derivatives, which reaches 73 cd m^−2^ afterglow brightness with a narrow FWHM of 24.2 nm.

## Graphical abstract

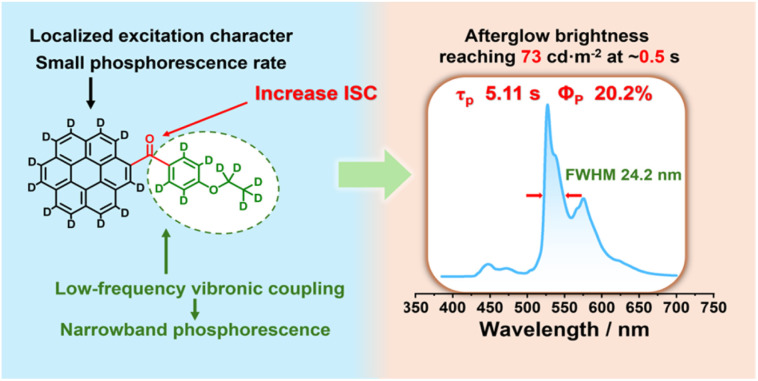

## Introduction

Organic afterglow materials hold great promise for advanced applications such as anticounterfeiting and information encryption, high-contrast bioimaging, and microenvironment sensing.^1–10^ The afterglow intensity is directly correlated with their performance and depends on multiple photophysical parameters, including the molar absorption coefficient (*ε*), afterglow quantum efficiency (*Φ*), and afterglow lifetime (*τ*), as expressed in the equation *L*(*t*) ∼ *εΦ*e^−*t/τ*^, where *L*(*t*) denotes materials' afterglow brightness at time *t*, after ceasing the excitation.^11–13^ In practice, the brightness of the afterglow largely dictates the suitability of a material for specific application scenarios.^14–25^ For instance, in thin-film devices, a high intrinsic brightness is essential to ensure a visible afterglow even at minimal thickness.^14,15^ In cases where micro- or nanoparticles are dispersed in media or physiological environments, their small dimension demands intrinsically bright emitters;^16–19^ otherwise, the afterglow signal becomes too weak to be practical. Similarly, emerging applications in illumination and display technologies require high-brightness emitters to meet operational demands.^20–23^ Moreover, in host–guest doping systems, bright emitters can function effectively at low doping concentrations, thereby lowering material cost and mitigating potential toxicity associated with the emitters.^24,25^

Recently, narrowband organic afterglow materials have emerged as a new class of luminescent materials, characterized by their small full width at half maximum (FWHM), typically below 40 nm or even 30 nm.^26–39^ Considering that the visible spectrum (400–700 nm) represents only a small fraction of the electromagnetic spectrum, the development of narrowband afterglow materials with a small FWHM is of great significance for maximizing the utilization of the visible region. Biomedical probes constructed from such materials enable multiplexed bioimaging with enhanced detection efficiency while maintaining a high signal-to-noise ratio.^26–30^ In advanced anticounterfeiting and information storage, narrowband afterglow materials can increase storage density and minimize spectral crosstalk, thereby improving the accuracy of information readout.^31–35^ Furthermore, their unique spectral characteristics are highly desirable for next-generation display technologies with wide color gamut, high color purity, and energy efficiency.^23,36–39^

In the literature, two main strategies have been reported for constructing organic narrowband afterglow materials.^29–35,39^ The first relies on energy transfer: a long-lived afterglow donor is paired with a spectrally matched narrowband fluorescent acceptor, yielding materials that can exhibit both long-lived afterglow and emission with a FWHM below 30 nm.^32^ This approach benefits from broad material choices and relatively simple synthesis, yet the final performance of the narrowband afterglow strongly depends on the energy transfer efficiency. The second strategy is based on the intrinsic molecular design of emitters, particularly through the development of multiple-resonance (MR) molecules.^29,30^ By tuning their radiative decay rate at room temperature, afterglow with FWHM below 40 nm can be achieved, and molecular cyclization can further reduce the FWHM.^33^ In these intrinsic systems, narrower afterglow can be realized by enhancing the MR effect; however, this inevitably shortens the lifetime, which is detrimental to the construction of high-brightness materials, as the afterglow intensity decreases exponentially with the reduction of lifetime according to the brightness equation.

To better understand the current state of the field, we conducted a statistical survey of high-performance afterglow and narrowband afterglow materials reported in the literature, summarizing their efficiencies and lifetimes, calculated *L*(*t*) and afterglow brightness at specific time after ceasing excitation (Table S1). The results clearly reveal that high-brightness organic afterglow materials are rare, and high-brightness organic narrowband afterglow materials are even scarcer, posing a major limitation for their practical applications. At present, therefore, the development of high-brightness narrowband afterglow materials remains a formidable challenge.

**Fig. 1:**
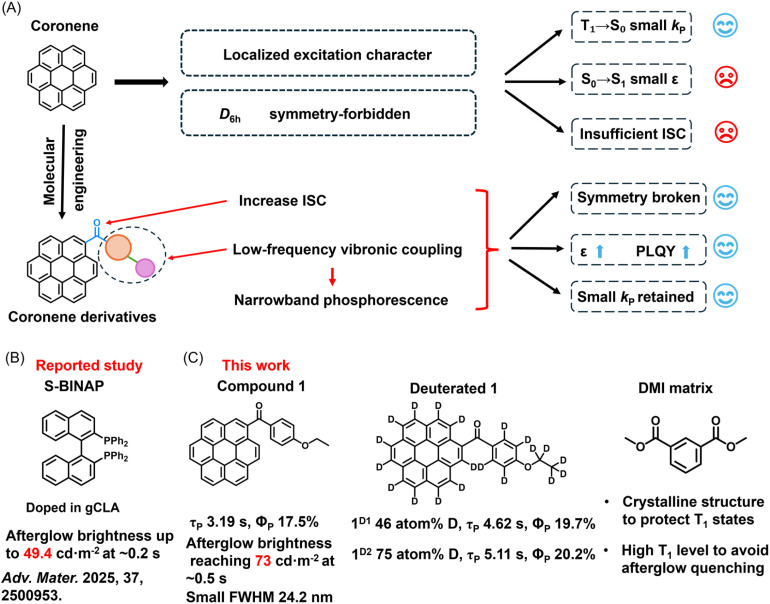
(A) Photophysical properties of coronene and molecular engineering to devise bright and narrowband phosphorescence in the coronene derivative system. (B) Selected examples of bright organic afterglow materials in the literature. (C) Ultrabright and narrowband organic afterglow materials obtained by doping the coronene derivative into the DMI matrix in this work.

We pioneered a low-frequency vibronic coupling strategy for devising intrinsic narrowband organic afterglow materials in localized excitation systems, where the materials can simultaneously possess a small FWHM and long phosphorescence lifetimes. Here we select coronene (Cor) as the luminescent core of localized excitation character. In coronene-based systems, the *T*_1_ → *S*_0_ transition is both spin-forbidden and symmetry-forbidden under *D*_6h_ symmetry, resulting in a small phosphorescence rate constant (*k*_P_).^40–44^ When the *T*_1_ state is well protected, these systems can exhibit phosphorescence lifetimes ranging from seconds to tens of seconds, making them excellent high-brightness emission centers. However, the *S*_0_ → *S*_1_ transition in coronene is symmetry-forbidden, leading to weak absorption in the 360–400 nm region (*vide infra*), and the intersystem crossing (ISC) efficiency remains insufficient (Fig. 1A). To address these limitations, we perform molecular engineering on coronene by introducing carbonyl groups (Fig. 1A). The n–π* character of carbonyls facilitates ISC, while their attachment to coronene breaks the molecular symmetry, thereby enhancing light absorption and improving the photoluminescence quantum yield (PLQY). Additional small substituents such as phenyl and alkoxy groups were introduced. These substituents possess higher *T*_1_ energy levels and thus do not significantly participate in the formation of the *T*_1_ state of coronene derivatives, allowing the *T*_1_ state to largely retain the intrinsic characteristics of the coronene core. Consequently, the *k*_P_ would remain small, and under appropriate matrix protection, coronene derivatives are capable of sustaining long phosphorescence lifetimes (*τ*_P_). Equally important, these small substituents would enrich the system with low-frequency vibrational modes that couple strongly with the *T*_1_ → *S*_0_ electronic transition (Fig. 1A). This vibronic coupling markedly influences the phosphorescence spectrum, which would lead to the emergence of narrowband emission features.

Building on the above design, we doped specifically engineered coronene derivatives into dimethyl isophthalate (DMI) matrices, affording narrowband afterglow materials with a phosphorescence efficiency of 17.5%, a phosphorescence lifetime of 3.19 s, and a FWHM of 24.2 nm under ambient conditions (Fig. 1C). Through experimental investigations combined with an analysis of low-frequency vibronic coupling, we established the structure–property relationship between coronene derivatives and their narrowband phosphorescent emission. Compared with the brightest organic afterglow materials reported in the literature (Fig. 1B 50 cd m^−2^ at ∼0.2 s after removing excitation source), our materials exhibit an afterglow brightness of 73 cd m^−2^ at ∼0.5 s after cessation of excitation. Upon deuteration of the coronene derivatives, the phosphorescence lifetime further increased to 5.11 s with a phosphorescence efficiency of 20.2%, leading to further enhancement of afterglow brightness (Fig. 1C). These findings not only deepen the mechanistic understanding of how vibronic coupling governs excited-state properties, but also underscore the significance of coronene derivatives as promising platforms for advancing the practical applications of organic afterglow materials across diverse fields.

## Results and discussion

**Fig. 2:**
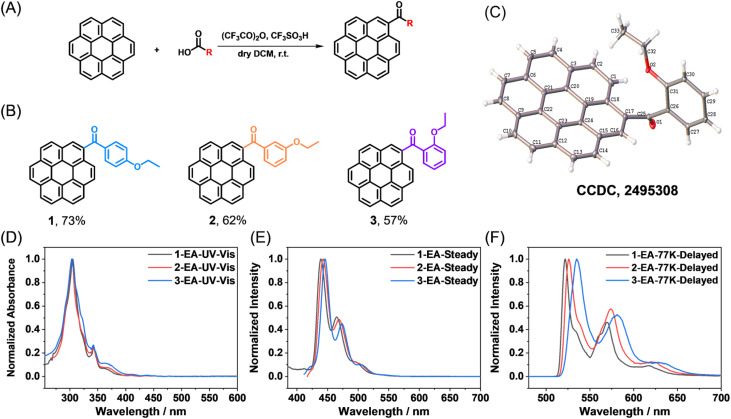
(A) Synthesis of coronene derivatives *via* acid-catalyzed Friedel–Crafts reaction. (B) Chemical structure of compounds 1, 2, and3, as well as the isolation yields. (C) Single crystal structure of compound 3. (D) UV-Vis absorption spectra of 1, 2, 3in ethyl acetate (EA) solution. (E) Steady-state spectra of compounds 1, 2, 3 in EA solution at room temperature. (F) Delayed emission (1 ms delay) spectra of 1, 2, 3 in EA solution at 77 K.

With the assistance of trifluoroacetic anhydride and trifluoromethanesulfonic acid, coronene derivatives (CoDe) were synthesized in a one-step reaction from aromatic carboxylic acids and coronene (Fig. 2A). After purification by column chromatography, the products were obtained in appreciable isolation yields of 57–73% (Fig. 2B), with their structures unambiguously confirmed by NMR (Fig. S33–35), mass spectrometry (Fig. S36–38), infrared spectroscopy (Fig. S39–41), and single-crystal X-ray diffraction (Fig. 2C and Table S3–8), and their high purity verified by HPLC (Fig. S1). Owing to the wide availability, structural diversity, and low cost of aromatic carboxylic acids, this synthetic route provides a facile and efficient strategy for accessing a broad library of coronene derivatives with minimal effort. In this work, we selected three isomeric coronene derivatives for detailed investigation. Each molecule consists of a coronene core, a carbonyl group directly attached to the coronene, a phenyl ring, and an ethoxy substituent, differing only in the substitution position of the ethoxy group on the phenyl ring. As will be demonstrated in the following sections, such a subtle structural variation leads to pronounced differences in photophysical behavior. Remarkably, the *para*-substituted ethoxy derivative exhibits exceptionally bright and narrowband afterglow emission in the DMI host.

We first examined the photophysical behavior of the coronene derivatives in solution. UV-Vis spectra recorded in typical organic solvents display intense absorption between 300 and 310 nm (molar absorption coefficients of 1.14–1.35 × 10^5^ M^−1^ cm^−1^), alongside prominent features spanning 330–380 nm (0.29–0.36 × 10^5^ M^−1^ cm^−1^) and a weak tail extending into the visible region (Fig. 2D and Table S2). The molar absorption coefficients of the coronene derivatives are much higher than that of coronene in the 350–400 nm region (Fig. S5), which suggest that the problem of symmetry-forbidden absorption of coronene was solved. Upon excitation, their steady-state emission presents fluorescence in the 420–550 nm range, peaking at 440–450 nm with a shoulder near 470 nm (Fig. 2E and S2–4). At 77 K, rigid solutions in ethyl acetate exhibit a bright greenish-yellow afterglow that persists for 23–30 s after the excitation is turned off (Fig. S6–11). Delayed emission spectra (1 ms delay) show structured phosphorescence bands peaked at around 530 nm (Fig. 2F). These results, along with long phosphorescence lifetimes ranging from 3.28 to 3.33 s (Fig. S6–11), are consistent with the predominantly localized excitation character of the *T*_1_ → *S*_0_ transition, as supported by TD-DFT calculations (Fig. S12–14). Remarkably, the phosphorescence bands of compound 1 feature extremely narrow widths, with FWHMs approaching 10 nm (Fig. 2F).

The three coronene derivatives show no room-temperature afterglow in solution upon photoexcitation. This is because, although abundant *T*_1_ states may be generated upon photoexcitation, the flexibility of intramolecular motions of the *T*_1_ states is significantly increased compared to rigid or solid-state environments, which facilitates rapid nonradiative deactivation and quenches room-temperature phosphorescence. Similarly, their pure solid forms also fail to exhibit afterglow at room temperature, as strong intermolecular π–π stacking in the neat state induces electronic coupling and quenches the afterglow. Our group, along with others, has demonstrated that dopant–matrix systems provide an effective method for afterglow material fabrication.^45–50^ Crystalline small molecules with low conjugation can serve as suitable hosts, in which the *T*_1_ states of the doped emitters are well protected.^51,52^ Importantly, such host matrices typically absorb only in the UVB (280–315 nm) and UVC (100–280 nm) regions, thereby avoiding interference with the UVA (315–400 nm) and visible absorption of the emitters.^11,53^ Moreover, their high *T*_1_ energy levels minimize the likelihood of accepting energy transfer from the emitters' *T*_1_ states, thus preventing significant degradation or quenching of the afterglow performance.^45,54^

**Fig. 3:**
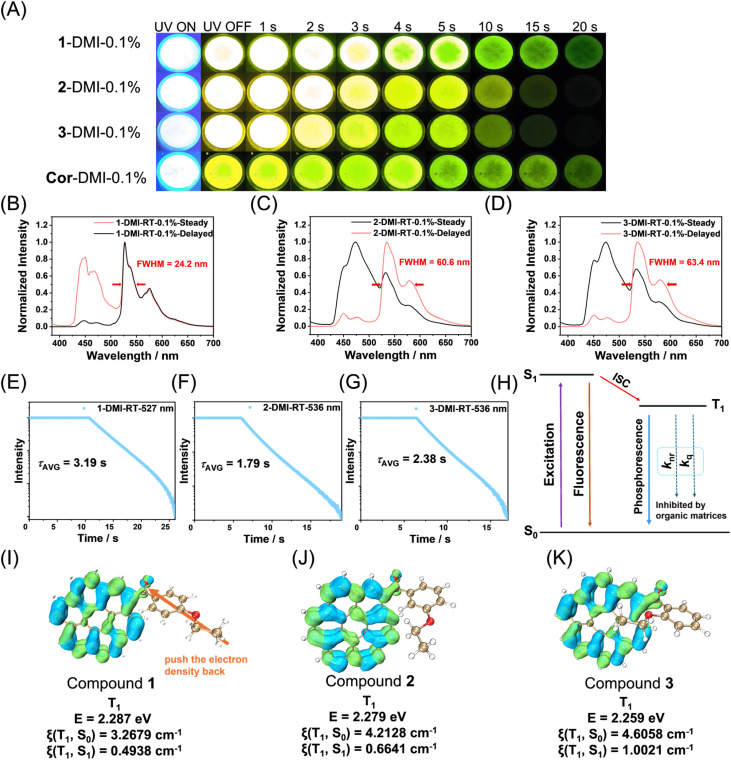
(A) Photograph of (1, 2, 3, Cor)-DMI-0.1% material afterglow object under UV lamp irradiation and after ceasing irradiation. (B–D) Room-temperature steady-state and delayed emission (1 ms delay) spectra of 1-DMI-0.1% (B), 2-DMI-0.1% (C), and 3-DMI-0.1% (D) samples. (E–G) Room-temperature emission decay profiles of 1-DMI-0.1% (E), 2-DMI-0.1% (F), and 3-DMI-0.1% (G) samples excited at 365 nm. (H) Schematic illustration of room-temperature phosphorescence mechanism in CoDe-DMI systems. (I–K) Isosurface diagram of the electron hole density difference in the *T*_1_ states of 1 (I), 2 (J), and 3 (K) in which the blue and green isosurfaces correspond to the hole and electron distribution, respectively.

We screened several small-molecule hosts, including phenyl benzoate (PhB), 4-methoxybenzophenone (MeOBP), phenyl 4-methoxybenzoate (MeOPhB), and dimethyl isophthalate (DMI). Among them, doping coronene derivatives into DMI produced the brightest room-temperature afterglow (Fig. S15–22). PhB, MeOPhB and DMI matrices have relatively higher *T*_1_ energy levels (3.49, 3.31 and 3.38 eV by TD-B3LYP/6-31g(d,p), respectively), which can prevent the afterglow quenching caused by excited state energy transfer from CoDe's *T*_1_ to matrices. In contrast, MeOBP has lower *T*_1_ energy level (2.89 eV by TD-B3LYP/6-31g(d,p)), so the afterglow performance of CoDe-MeOBP is inferior (Fig S17 and 18). Besides, DMI has two ester groups (for improved intermolecular interactions between the dopant and matrix) which would be useful for the dispersion of CoDe molecules to minimize aggregation-caused afterglow quenching. Therefore, DMI performs significantly better than the other screened hosts. With a melting point of 64–68 °C, DMI is readily processable by melt-casting, enabling the facile preparation of dopant–matrix afterglow materials. In subsequent studies, therefore, DMI was chosen as the host, with a typical doping concentration of 0.1% (Fig. S23 and 24). The CoDe-DMI-0.1% samples displayed bright room-temperature afterglow under 365 nm UV excitation, with durations of 16–32 s. Their afterglow brightness within 0–5 s after ceasing excitation was significantly higher than that of Cor-DMI-0.1%, with the 1-DMI-0.1% sample being the brightest (Fig. 3A). Notably, efficient afterglow was also observed upon 385 nm and 405 nm excitation (Fig. S25–28). The steady-state spectra of CoDe-DMI-0.1% at room temperature exhibited two emission bands in 420–500 and 500–700 nm regions, attributable to fluorescence and phosphorescence, respectively (Fig. 3B and D). The delayed spectra (1 ms delay) were dominated by a 500–700 nm phosphorescence band with distinct vibronic fine structures, including a main peak and shoulder peaks, consistent with *T*_1_ emission of coronene derivatives with localized excitation (LE) character. Interestingly, the phosphorescence spectrum of the 1-DMI-0.1% sample exhibits a small FWHM of 24.2 nm/846 cm^−1^ at room temperature and an exceptionally small FWHM of 5.6 nm/203 cm^−1^ at 77 K (Fig. S22). The phosphorescence lifetimes of CoDe-DMI-0.1% reached 1.79–3.19 s (Fig. 3E–G), corresponding to the yellow-green afterglow observable by the naked eye (Fig. 3A). It is worth noting that DMI exhibits negligible absorption at 365, 385, and 405 nm (Fig. S29), indicating that the observed afterglow is not derived from matrix-to-dopant energy transfer.^55^ With low conjugation, electron-inert characteristics, a deep HOMO, and a high LUMO, DMI is also unlikely to form intermolecular charge-transfer (CT) states with CoDe (Fig. S30).^56^ This is further supported by the fine-structured fluorescence spectrum of CoDe-DMI, in contrast to the broad emission typical of CT states. In addition, both CoDe and DMI were carefully purified, and their high purity was confirmed by HPLC (Fig. S1), excluding the possibility of impurity-induced afterglow.^57^ Taken together, the similarity between the room-temperature delayed spectra of the dopant–matrix materials (Fig. 3B–D) and the 77 K phosphorescence spectra of coronene derivatives in solution (Fig. 2F) confirms that the room-temperature afterglow originates from the *T*_1_-state phosphorescence of the coronene derivatives (Fig. 3H). Under 365 nm, 385 nm or visible-light excitation, the coronene derivatives absorb photons to populate the singlet excited state, undergo intersystem crossing to the triplet state, and, within the crystalline DMI host, experience suppressed nonradiative decay and oxygen quenching. The subsequent *T*_1_ → *S*_0_ transition generates phosphorescence (Fig. 3H). Owing to the LE character of the *T*_1_ state in coronene derivatives (Fig. 3I–K), the radiative rate constant (*k*_P_) remains small, resulting in long phosphorescence lifetimes and bright, persistent afterglow visible to the naked eye.

To elucidate the origin of the high brightness of the 1-DMI-0.1% sample, we first measured the PLQYs of the three samples, which were 36.2%, 30.3%, and 32.5%, respectively. In all cases, the steady-state spectra at room temperature displayed a substantial phosphorescence contribution, consistent with the role of carbonyl substituents in promoting intersystem crossing (ISC) in the CoDe systems. The phosphorescence contributions were 48.3%, 35.0%, and 37.8%, from which the corresponding phosphorescence efficiencies were calculated as 17.5% for 1-DMI-0.1%, exceeding those of 2-DMI-0.1% (10.6%) and 3-DMI-0.1% (12.3%). Why does 1-DMI-0.1% exhibit the highest phosphorescence efficiency? To address this, we examined its fluorescence radiative rate constant (*k*_F_) and singlet–triplet energy gap (Δ*E*_ST_). Using the relationship *k*_F_ = *φ*_F_/*τ*_F_, *k*_F_ of 1-DMI-0.1% was calculated to be 1.72 × 10^7^ s^−1^, lower than those of the other two samples (1.97 × 10^7^ s^−1^ and 2.51 × 10^7^ s^−1^, respectively) (Table 1 and Fig. S31). In the delayed spectra at room temperature, the phosphorescence peak of 1-DMI-0.1% appeared at 527 nm (corresponding to a *T*_1_ energy of 2.35 eV), which is shorter in wavelength than those of the other samples (536 nm, 2.31 eV). These experimental results are in good agreement with TD-DFT calculations of the relative *T*_1_ energy levels of the CoDe derivatives. In contrast, the *S*_1_ energy levels of the three CoDe-DMI samples were similar (∼2.75 eV), making the Δ*E*_ST_ of 1-DMI-0.1% the smallest (0.40 eV). Both the reduced *k*_F_ and the minimized Δ*E*_ST_ facilitate ISC, which accounts for the superior phosphorescence efficiency of 1-DMI-0.1% (Table 1).

**Table 1:** Photophysical properties of the CoDe-DMI materials

Samples	*λ*_P_/nm	FWHM	*τ*_p_/s	*Φ*_P_/%	*Φ*_F_/%	*τ*_F_/ns	*k*_F_/10^7^ s^−1^
1	527	24.2	3.19	17.5	18.7	10.86	1.72
2	536	60.6	1.79	10.6	19.7	10.02	1.97
3	536	63.4	2.38	12.3	20.2	9.36	2.16

Next, we compared the room-temperature phosphorescence lifetimes of the CoDe-DMI samples and analyzed the underlying reasons. The 1-DMI-0.1% sample exhibited a phosphorescence lifetime of 3.19 s under ambient conditions, longer than those of the 2- and 3-DMI systems (Fig. 3E–G). Examination of the TD-DFT-calculated *T*_1_ excited states revealed that, in addition to the LE character primarily localized on the coronene moiety, the carbonyl group also contributes to the *T*_1_ electron–hole distribution (Fig. 3I–K). Notably, in the 2- and 3-DMI systems, the carbonyl participation is greater than in 1-DMI, resulting in increased conjugation, consistent with the experimentally and computationally observed lower *T*_1_ energy levels for 2- and 3-DMI relative to 1-DMI. The higher carbonyl involvement in the *T*_1_ states of 2- and 3-DMI also enhances the spin–orbit coupling matrix elements (SOCME) for the *T*_1_ → *S*_0_ transition (Fig. 3I–K), leading to increased phosphorescence rate constants (*k*_P_). In contrast, the *T*_1_ state of 1-DMI exhibits a smaller *k*_P_, accounting for its longer room-temperature phosphorescence lifetime. Structurally, the *para*-substituted ethoxy group on the phenyl ring of compound 1 exerts electron-donating effects that push the *T*_1_-state electron density, originally localized on the carbonyl, back onto the coronene LE state (Fig. 3I). This reduces *k*_P_ and prolongs the phosphorescence lifetime. This analysis provides a clear understanding of the structure–phosphorescence relationship in coronene derivatives. The strong agreement between experimental observations and theoretical calculations not only validates the accuracy of our measurements but also confirms the reliability of the computational approach.

**Fig. 4:**
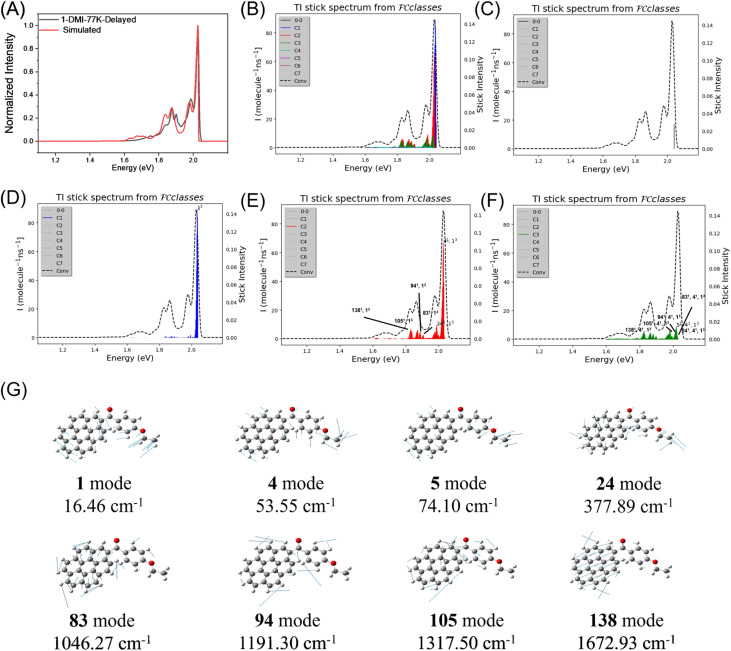
(A) Experimental (black) and simulated (red) phosphorescence spectra of compound 1; the experimental phosphorescence spectrum was shifted for ease of comparison. (B–F) Simulated spectra by Franck–Condon analysis and the corresponding stick spectra for compound 1. In the spectra, the vibronic bands are assigned as *X*^*y*^, where *X* denotes the oscillator and *y* represents its quantum number. (G) The vibrations of 1 involved in the Franck–Condon spectral progression at the B3LYP/6-31g(d,p) theoretical level.

Based on the detailed analysis of phosphorescence efficiency and lifetime, the exceptional afterglow brightness of 1-DMI-0.1% can be rationalized. Another noteworthy photophysical feature of this material is its narrowband emission, characterized by a small FWHM of 24.2 nm/846 cm^−1^ at room temperature and 5.6 nm/203 cm^−1^ at 77 K (Fig. S22), which presented a significant challenge during our investigation. To elucidate the origin of this narrowband phosphorescence, the *T*_1_-state geometry was first optimized at the B3LYP/6-31g(d,p) level using Gaussian 16, followed by vibrational frequency calculations for both the *T*_1_ and *S*_0_ states. Phosphorescence spectra were then simulated using FCClasses 3.0.^58^ The calculated spectra showed excellent agreement with the experimental results for 1-DMI materials in terms of spectral shape (Fig. 4A). For compound 1, phosphorescence arises predominantly from the C_1_ and C_2_ vibronic classes, with less contribution from the 0–0 transition and C_3_ class (Fig. 4B–F). Notably, within the C_1_ and C_2_ classes, low-frequency vibronic bands were considerably more intense than the high-frequency ones, giving rise to the observed narrowband emission (Fig. 4D and E). The experimental results and theoretical analysis shed light on the fundamental behavior of vibronic coupling in the coronene derivative systems. In coronene-based systems, *k*_P_ is generally small.^25,40–44,46,53,59,60^ As a result, the 0–0 transition contributes minimally to the phosphorescence spectrum, allowing low-frequency vibrational modes coupled with the *T*_1_ → *S*_0_ electronic transition to exert a significant influence on the spectral profile (Fig. 4G). Based on our previous studies, *para*-substituted functional groups on the benzoyl moiety can further participate in the formation of low-frequency vibrational couplings in the C_n_ class. In the present 1-DMI system, the *para*-substituted ethoxy group plays precisely this role, enhancing the low-frequency vibrational coupling (Fig. 4G). Consequently, the phosphorescence peaks arising from these low-frequency vibronic couplings dominate over signals from high-frequency vibrational couplings, leading to the observed narrowband phosphorescence with a small FWHM in the 1-DMI system. Compared to the relatively large FWHMs in 2-DMI and 3-DMI systems, one can find that the *para*-substitution of a small functional group is very useful for the achievement of a small FWHM in the CoDe system. This study, together with the on-going studies in our research group, reveals that the symmetry of *para*-substitution can generate a rich low-frequency vibrational mode in the CoDe system; such a low-frequency vibrational mode can couple with the *T*_1_-to-*S*_0_ electronic transition and participate in the formation of CoDe's phosphorescence spectra, giving rise to narrowband emission. This finding would inspire the molecular design of the CoDe system in our future study.

In contrast to the multiple-resonance design principle that minimizes high-frequency vibronic coupling, our strategy focuses on enhancing the low-frequency vibronic coupling to attain narrowband luminescence. In luminescent materials where emission originates primarily from localized excited states, the spectrum typically consists of multiple narrow vibronic bands. Although each individual band is sharp, their superposition usually produces a broad emission spectrum with a large full width at half maximum. We demonstrate that narrowing the overall emission could be achieved by modulating the relative intensities of these vibronic bands—specifically, by increasing the low-frequency vibronic contributions relative to the high-frequency ones, such that their intensity ratio becomes above 2. The present strategy avoids the side effect in multiple-resonance systems (the enhancement of the multiple-resonance effect leads to spectral narrowing but emission lifetime decrease), and thus makes the achievement of long phosphorescence lifetime and small FWHM in the intrinsic narrowband emission system possible.

**Fig. 5:**
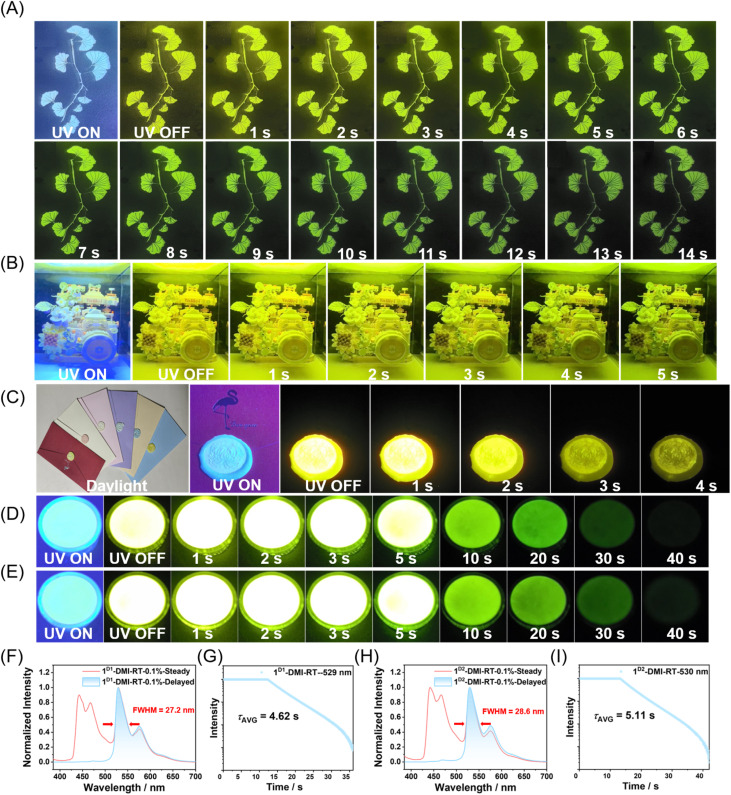
(A) Photographs of the gingko leaf-shaped afterglow objects of 1-DMI materials under and after the removal of the 365 nm UV light. (B) Photograph of a camera block (17 × 17 × 17.5 cm) illuminated by an afterglow plate (20 × 20 cm) made from the 1-DMI-0.1% material, showing the state with the excitation light source on and off. (C) Photographs of 1-DMI-0.1% mixed with different colored wax seals, showing the state when the excitation source is turned on and off. (D) Photograph of the 1^D1^-DMI-0.1% material afterglow object. (E) Photograph of the 1^D2^-DMI-0.1% material afterglow object. Steady-state and delayed emission (1 ms delay) spectra and phosphorescence decay of 1^D1^-DMI-0.1% (F and G) and 1^D2^-DMI-0.1% (H and I) samples at room temperature (excited at 365 nm).

The 1-DMI system exhibits long-lived afterglow with both high brightness and small FWHM, offering broad potential in applications such as displays, anticounterfeiting, and information encryption. By sandwiching molten 1-DMI-0.1% between two glass panels, we fabricated afterglow panels of 20 cm × 20 cm. When a patterned frame was placed on the panel and the excitation light source removed, the pattern remained clearly visible, demonstrating the potential of these panels for illumination purposes. Placing a ginkgo leaf pattern on the panel revealed a bright and high-color-purity image, highlighting the aesthetic appeal and practical utility of these materials in display applications (Fig. 5A). When the afterglow plate was further applied to illuminate a camera block (17 × 17 × 17.5 cm), the object was brightly visible even after the excitation light was turned off, demonstrating the high brightness and strong illumination capability of the material for practical lighting applications (Fig. 5B). Furthermore, this afterglow material is well-suited for anticounterfeiting and information encryption. By melting the afterglow sample with paraffin at 70 °C and applying the mixture to a sealed envelope, patterned stamps (*e.g.*, a chrysanthemum motif) can be pressed onto the hot sample. Upon cooling at room temperature, the material solidifies, sealing the envelope. Under UV illumination, the seal exhibits strong fluorescence, and upon turning off the light, a bright yellow-green afterglow reveals the chrysanthemum pattern (Fig. 5C). This simple approach enables high-level anticounterfeiting and secure information encoding with minimal effort.

Finally, we considered that molecular deuteration could further enhance room-temperature afterglow properties. This is because the C–D vibrational modes in deuterated molecules are significantly weaker than the corresponding C–H vibrations in non-deuterated analogues, resulting in reduced nonradiative decay from the *T*_1_ state.^18,45^ When deuterated molecules are well protected within a host matrix, brighter room-temperature afterglow can be achieved. We deuterated compound 1 to an average deuteration level of 46 atom% D (compound 1^D1^), and doping this into the DMI host produced a material whose afterglow brightness and duration under UV excitation were visibly enhanced (Fig. 5D). In the phosphorescence decay curve, the intensity remained saturated (the Hitachi F-4700 instrument limits the intensity to 10 000 to protect the detector) from 0 to 13 s, and only after 13 s did normal phosphorescence decay become observable (Fig. 5G). Fitting yielded an average phosphorescence lifetime of 4.62 s, longer than that of the non-deuterated system, demonstrating the significant effect of deuteration on afterglow performance. Further increasing the deuteration level of compound 1 to obtain compound 1^D2^ (75 atom% D), the resulting dopant–matrix material exhibited an extended phosphorescence lifetime of 5.11 s, a PLQY of 42.9%, and a phosphorescence efficiency of 20.2% calculated from the integrated steady-state spectrum. The material displayed narrowband emission with a FWHM of 28.6 nm, representing unprecedented performance among organic afterglow systems. TD-DFT calculation for the deuterated compound 1 reveals that, for the *S*_1_-*T*_1_ and *S*_1_-*T*_2_ ISC channels, the deuterated compound 1 has larger spin–orbit coupling matrix elements (SOCMEs) than pristine compound 1 (Fig. S32). The SOCME of *T*_1_–*S*_0_ transition (related to phosphorescence rate) for the deuterated compound 1 is smaller than that of pristine compound 1. On the other hand, deuteration of polycyclic aromatic hydrocarbons would decrease the energy of effective nonclassical vibrational modes due to the weaker C–D stretching compared to C–H stretching. This reduces the Franck–Condon factor involved in nonradiative transitions, thereby decreasing the nonradiative decay rate.^18,45^ The above results and analysis can explain the higher phosphorescence efficiency and longer phosphorescence lifetime in the deuterated system. In the present case, the deuteration of compound 1 has an insignificant impact on the shape of phosphorescence spectra and the FWHM value. Moving forward, we will explore the functional applications of such materials in anticounterfeiting, information storage, bioimaging, and oxygen sensing.

## Conclusion

In summary, we have developed a low-frequency vibronic coupling strategy that enables the molecular design of ultrabright and narrowband organic afterglow materials. By introducing aroyl and ethoxy substituents into coronene, we broke its molecular symmetry, enhanced light absorption and intersystem crossing, and preserved a localized triplet state with a small radiative rate constant. The optimized CoDe-DMI system exhibits an exceptional combination of high phosphorescence efficiency (up to 20.2%), long lifetime (5.11 s), and remarkably narrow emission bandwidth < 30 nm. Theoretical analyses reveal that the spectral narrowing originates from dominant low-frequency vibronic coupling within the coronene derivative system, which governs the fine vibronic structure of the phosphorescence. This work not only provides a fundamental understanding of vibronic coupling–controlled afterglow processes, but also establishes a generalizable molecular-engineering principle for creating next-generation organic afterglow materials. The outstanding brightness and color purity achieved herein open promising avenues for illumination, display, anticounterfeiting, and information encryption technologies.

## Author contributions

Y. C. carried out the data curation, formal analysis, investigation, and validation. Y. Z. created the methodology. G. W., T. L., J. J. and T. W. supported the work through photophysical measurements and software. Y. C and K. Z. wrote and edited the original manuscript. K. Z. and X. G. led the project supervision and conceptualization.

## Conflicts of interest

The authors declare no competing financial interest.

## Supplementary Material

SC-017-D5SC08966B-s001

SC-017-D5SC08966B-s002

## Data Availability

The data supporting this article have been included as part of the supplementary information (SI). Supplementary information: experimental procedures, computational studies, and characterization data for the new compounds. See DOI: https://doi.org/10.1039/d5sc08966b. CCDC 2495308 (3) contains the supplementary crystallographic data for this paper.^61^
