# Supplementary material for: Ultrabright and narrowband organic afterglow achieved by molecular engineering of coronene
Source: Chem Sci. 2026 Jan 9;17(9):4538–47. doi: 10.1039/d5sc08966b (PMC12809253; doi:10.1039/d5sc08966b)
Supplement: SC-017-D5SC08966B-s001 [file SC-017-D5SC08966B-s001.pdf]

Supplementary Information (SI) for Chemical Science.
This journal is © The Royal Society of Chemistry 2026

Supplementary Information

Ultrabright and narrowband organic afterglow achieved by molecular engineering of coronene

Yuanyuan Chen,[a,b] Yue Zhang,[b] Guoyi Wu,[b] Ting Luo,[b] Jialiang Jiang,[b] Tengyue Wang,[b] Xiaoya Guo,*[a] Kaka Zhang*[b]

[a]Department of Chemical Engineering, Shanghai University, Shanghai, 200444, China. Email: gxy@shu.edu.cn;

[b]State Key Laboratory of Organometallic Chemistry and Shanghai-Hong Kong Joint Laboratory in Chemical Synthesis, Key Laboratory of Synthetic and Self-Assembly Chemistry for Organic Functional Molecules, Ningbo Zhongke Creation Center of New Materials, Shanghai Institute of Organic Chemistry, University of Chinese Academy of Sciences, Chinese Academy of Sciences, 345 Lingling Road, Shanghai 200032, People's Republic of China. E-mail: zhangkaka@sioc.ac.cn.

Table of Contents

Materials
Physical measurements and instrumentation
Computational methods
Synthesis of coronen-1-yl(4-ethoxyphenyl) methanone (Compound 1)
Synthesis of coronen-1-yl(3-ethoxyphenyl) methanone (Compound 2)
Synthesis of coronen-1-yl(2-ethoxyphenyl) methanone (Compound 3)
Synthesis of deuterated compound 1
Table S1. Selected examples of organic afterglow materials with corresponding lifetimes and afterglow efficiency under ambient conditions.
Table S2. Photophysical properties of the coronene derivatives in dichloromethane (DCM), toluene (PhMe) and ethyl acetate (EA): absorption peaks (λ_{abs}), molar absorption coefficients (ε), and fluorescence maxima (λ_F) (excited at 365 nm).
Figure S1. HPLC profile of compound **1-3** and DMI.
Figure S2. UV-Vis absorption spectra (normalized) and the steady-state emission spectra (excited at 365 nm) of coronene derivatives in dichloromethane (DCM) solution at room temperature.
Figure S3. UV-Vis absorption spectra (normalized) and the steady-state emission spectra (excited at 365 nm) of coronene derivatives in EA solution at room temperature.
Figure S4. UV-Vis absorption spectra (normalized) and the steady-state emission spectra (excited at 365 nm) of coronene derivatives in toluene (PhMe) solution at room temperature.
Figure S5. UV-Vis spectrum of coronene in dichloromethane. Inset shows the weak absorption in the range of 350-400 nm. (**b**) UV-vis spectra of coronene and **CoDe.**
Figure S6. Afterglow photograph of **1**-EA solution at 77 K.
Figure S7. (**a**) Steady-state and delayed emission (1 ms delay) spectra and (**b**) phosphorescence decay of compound **1** in EA solution at 77 K (excited at 365 nm).
Figure S8. Afterglow photograph of **2**-EA solution at 77 K.

Figure S9. (**a**) Steady-state and delayed emission (1 ms delay) spectra and (**b**) phosphorescence decay of compound **2** in EA solution at 77 K (excited at 365 nm).

Figure S10. Afterglow photograph of **3**-EA solution at 77 K.

Figure S11. (**a**) Steady-state and delayed emission (1 ms delay) spectra and (**b**) phosphorescence decay of compound **3** in EA solution at 77 K (excited at 365 nm).

Figure S12. Iso-surface maps of electron-hole density difference of **1**'s S_n and T_n states, where blue and green iso-surfaces correspond to hole and electron distributions, respectively, and SOCME values. The ground-state geometry was optimized by a DFT calculation using B3LYP functional and 6-31g(d,p) basis set. The singlet excited states and triplet excited states were calculated on Gaussian 16 program (Revision A.03) with B3LYP functional and 6-31g(d,p) functional. Spin-orbit coupling (SOC) matrix elements between the singlet excited states and triplet excited states were calculated with spin-orbit mean-field (SOMF) methods on ORCA 5.0.3 program with B3LYP functional and 6-31g(d,p) basis set.

Figure S13. Iso-surface maps of electron-hole density difference of **2**'s S_n and T_n states, where blue and green iso-surfaces correspond to hole and electron distributions, respectively, and SOCME values. The ground-state geometry was optimized by a DFT calculation using B3LYP functional and 6-31g(d,p) basis set. The singlet excited states and triplet excited states were calculated on Gaussian 16 program (Revision A.03) with B3LYP functional and 6-31g(d,p) functional. Spin-orbit coupling (SOC) matrix elements between the singlet excited states and triplet excited states were calculated with spin-orbit mean-field (SOMF) methods on ORCA 5.0.3 program with B3LYP functional and 6-31g(d,p) basis set.

Figure S14. Iso-surface maps of electron-hole density difference of **3**'s S_n and T_n states, where blue and green iso-surfaces correspond to hole and electron distributions, respectively, and SOCME values. The ground-state geometry was optimized by a DFT calculation using B3LYP functional and 6-31g(d,p) basis set. The singlet excited states and triplet excited states were calculated on Gaussian 16 program (Revision A.03) with B3LYP functional and 6-31g(d,p) functional. Spin-orbit coupling (SOC) matrix elements between the singlet excited states and triplet excited states were calculated with spin-orbit mean-field (SOMF) methods on ORCA 5.0.3 program with B3LYP functional and 6-31g(d,p) basis set.

Figure S15. Photograph of **1**-PhB-0.1% material afterglow object.

Figure S16. (**a**) Steady-state and delayed emission (1 ms delay) spectra and (**b**) phosphorescence decay of **1**-PhB-0.1% samples at room temperature (excited at 365 nm).

Figure S17. Photograph of **1**-MeOBP-0.1% material afterglow object.

Figure S18. (**a**) Steady-state and delayed emission (1 ms delay) spectra and (**b**) phosphorescence decay of **1**-MeOBP-0.1% samples at room temperature (excited at 365 nm).

Figure S19. Photograph of **1**-MeOPhB-0.1% material afterglow object.

Figure S20. (**a**) Steady-state and delayed emission (1 ms delay) spectra and (**b**) phosphorescence decay of **1**-MeOPhB-0.1% samples at room temperature (excited at 365 nm).

Figure S21. Photograph of **1**-DMI-0.1% material afterglow object.

Figure S22. (**a**, **b**) Room temperature and (**c**, **d**) 77 K steady-state, delayed emission spectra (1 ms delay) and phosphorescence decay profiles of **1**-DMI-0.1% samples (excited at 365 nm).

Figure S23. Photograph of **1**-DMI-(0.01%, 0.1%, 1%) samples.

Figure S24. Room temperature steady-state, delayed emission spectra (1 ms delay) and phosphorescence decay profiles of **1**-DMI-(0.01%, 0.1%, 1%) samples (excited at 365 nm). The

afterglow brightness of these samples is similar at different concentrations (Fig. S22). The 1-DMI-0.1% sample shows a smaller FWHM than 1-DMI-0.01% and 1-DMI-1%. Therefore, we choose 0.1% doping concentration in this study.

Figure S25. Photograph of **1**-DMI-0.1% material afterglow object (excited at 385 nm).

Figure S26. (a) Steady-state and delayed emission (1 ms delay) spectra and **(b)** phosphorescence decay of **1**-DMI-0.1% samples at room temperature (excited at 385 nm).

Figure S27. Photograph of **1**-DMI-0.1% material afterglow object (excited at 405 nm).

Figure S28. (a) Steady-state and delayed emission (1 ms delay) spectra and **(b)** phosphorescence decay of **1**-DMI-0.1% samples at room temperature (excited at 405 nm).

Figure S29. (a) Normalized UV-vis absorption spectra, **(b)** steady-state and delayed emission spectra (excited at 365 nm) of DMI in n-hexane solution at 77K.

Figure S30. HOMO/LUMO energy levels of DMI, coronene and its derivatives calculated at B3LYP/6-31g(d,p).

Figure S31. (a) The prompt fluorescence decay profiles of **1**-DMI-0.1% sample excited at 365 nm and monitored at 450 nm. **(b)** The prompt fluorescence decay profiles of **2**-DMI-0.1% sample excited at 365 nm and monitored at 474 nm. **(c)** The prompt fluorescence decay profiles of **3**-DMI-0.1% sample excited at 365 nm and monitored at 471 nm.

Figure S32. Iso-surface maps of electron-hole density difference of deuterated compound **1**'s S_n and T_n states, where blue and green iso-surfaces correspond to hole and electron distributions, respectively, and SOCME values. The ground-state geometry was optimized by a DFT calculation using B3LYP functional and 6-31g(d,p) basis set. The singlet excited states and triplet excited states were calculated on Gaussian 16 program (Revision A.03) with B3LYP functional and 6-31g(d,p) functional. Spin-orbit coupling (SOC) matrix elements between the singlet excited states and triplet excited states were calculated with spin-orbit mean-field (SOMF) methods on ORCA 5.0.3 program with B3LYP functional and 6-31g(d,p) basis set.

Figure S33. ^{1}H NMR of compound **1** (400 MHz, chloroform-d, 298 K).

Figure S34. ^{1}H NMR of compound **2** (400 MHz, chloroform-d, 298 K).

Figure S35. ^{1}H NMR of compound **3** (400 MHz, chloroform-d, 298 K).

Figure S36. HRMS of compound **1**.

Figure S37. HRMS of compound **2**.

Figure S38. HRMS of compound **3.**

Figure S39. FT-IR spectra of compound **1**.

Figure S40. FT-IR spectra of compound **2**.

Figure S41. FT-IR spectra of compound **3**.

Table S3. Crystal data and structure refinement for compound **3**.

Table S4. Atomic coordinates (x 10^4) and equivalent isotropic displacement parameters ($Å^2$x 10^3) for mo_d8v25343_0m. U(eq) is defined as one third of the trace of the orthogonalized U^{ij} tensor.

Table S5. Bond lengths [Å] and angles [°] for compound **3**.

Table S6. Anisotropic displacement parameters ($Å^2$x 10^3) for compound **3**. The anisotropic displacement factor exponent takes the form: $-2\pi^2$ [h^2 $a^{*2}U^{11}$ + ... + 2 h k a* b* U^{12}]

Table S7. Hydrogen coordinates (x 10^4) and isotropic displacement parameters ($Å^2$x 10 ^{3}) for compound **3**.

Table S8. Torsion angles [°] for compound **3**.

Supplementary References

Materials

Coronene (RG, Adamas), 4-ethoxybenzoic acid (98%, Heowns Biochem), 3-ethoxybenzoic acid (RG, Adamas), 2-ethoxybenzoic acid (97%, Bio-pharmaceutical), anhydrous dichloromethane (99.9%, J&K Scientific), trifluoroacetic anhydride (TFAA, 98%, Adamas), trifluoromethanesulfonic acid (TFOH, RG, Adamas), 1,4-dioxane (AR, Acmec), dimethyl isophthalate (DMI, 99%RG, Adamas), phenyl benzoate (PhB, 99%, Energy Chemical), 4-methoxybenzophenone (MeOBP, 98%, Bio-pharmaceutical), toluene (PhMe, AR, Sinopharm), dicholoromethane (DCM, AR, Sinopharm), ethyl acetate (EA, AR, Sinopharm), deuterium oxide (D_2O, RG, Adamas), phenyl 4-methoxybenzoate (MeOPhB, 98%, J&K Scientific), chloroform-d (99.8%, J&K Scientific).

Physical measurements and instrumentation

Nuclear magnetic resonance (NMR) spectra were recorded on JEOL Fourier-transform NMR spectrometer (400 MHz). FT-IR spectra were recorded on a Nicolet AVATAR-360 FT-IR spectrophotomerter with a resolution of 4 cm^{-1}. The X-ray crystallographic data for the compound has been deposited at the Cambridge Crystallographic Data Centre (CCDC). Single-crystal X-ray diffraction analysis was performed on a Bruker D8 Venture diffractometer. The crystal was kept at 143.0 K during data collection. Using Olex2, the structure was solved with the SHELXT structure solution program using Intrinsic Phasing and refined with the SHELXL refinement package using Least Squares minimisation. High-resolution mass spectrometry (HRMS) analyses were performed by Agilent Technologies 5973N and Thermo Fisher Scientific LTQ FT Ultra mass spectrometer operated in positive electrospray ionization (ESI) mode. HPLC measurement was performed on a waters ACQUITY UPLC H-Class system by using MeCN/DCM (volume ratio 2:1) as eluent. UV-Vis absorption spectra were recorded on a Techcomp UV1050 UV-vis spectrophotometer and Shimadzu UVmini1285 UV-vis spectrophotometer. The steady-state and delayed emission spectra were collected by Hitachi F-4700 fluorescence spectrometer equipped with chopping systems; the delayed emission spectra were obtained with a delay time of approximately 1 ms. The brightness of the afterglow was measured using the Digital Lux Meter GM1040. The fluorescence decay profiles in nanosecond region were recorded by using time-correlated single photon counting technique (TCSPC) on an Edinburgh FLS1000 fluorescence spectrometer equipped with a picosecond pulsed diode laser. Photoluminescence quantum yield was measured by a Hamamatsu absolute PL quantum yield measurement system based on a standard protocol. Photographs and videos were captured by HUAWEI Mate 60 pro cameras. Before the capture, samples were irradiated by a 365 nm UV lamp (5 W) for approximately 2 s at a distance of approximately 15 cm.

Computational methods

For TD-DFT calculation, the ground-state geometries of compounds **1-3,** dimethyl isophthalate (DMI) and coronene were optimized at the B3LYP/6-31g(d,p) level of theory, followed by frequency analysis at the same level. No imaginary modes were obtained. The highest occupied molecular orbital (HOMO) and lowest unoccupied molecular orbital (LUMO) were computed at B3LYP/6-31g(d,p). All of the calculations mentioned above were performed by Gaussian 16 package.[1] Excitation energy, spin-orbit coupling matrix elements (SOCME) were calculated at the B3LYP/G/def2-TZVP(-f) level of theory with spin-orbit mean-field (SOMF)

methods on ORCA 5.0.3 program.[2,3] All iso-surface maps to show the electron distribution and electronic transitions were rendered by Visual Molecular Dynamics (VMD 1.9.3) software [4] based on the exported files from Multiwfn 3.8.[5,6]

For the phosphorescence emission spectra simulation, the excited-state (T_1) geometries of compounds were optimized at B3LYP/6-31g(d,p) level by Gaussian 16 package. The frequency analysis was performed for both the T_1 and S_0 based on the optimized T_1 geometries using Gaussian 16 package. The phosphorescence emission spectra simulation by Franck-Condon analysis and stick spectra were computed by FCclasses 3.[7] The .inp file for FCclasses 3 is :

```
$$$
PROPERTY = EMI;
MODEL = VH;
DIPOLE = FC;
TEMP = 0;
; DE = (READ);
BROADFUM = GAU;
HWHM = 0.001;
METHOD = T1;
;VIBRATIONAL ANALYSIS
NORMALMODES = COMPUTE;
COORDS = INTERNAL;
;INPUT DATA FILES
STATE1_FILE = T1.fcc
STATE2_FILE = S0.fcc
ELDIP_FILE = eldip_T1TD_fchk
```

And all compounds computed at the same theoretical level. The output spectrum data yield a HWHM value of 0.01.

Synthesis of coronen-1-yl(4-ethoxyphenyl) methanone (Compound 1)

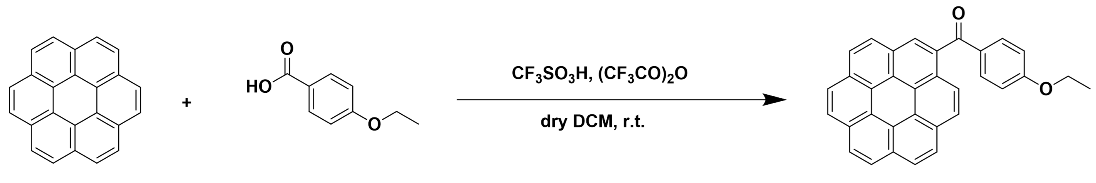

Coronene **1**

4-Ethoxybenzoic acid (66.5 mg, 0.4 mmol, 1.3 equiv.) and anhydrous dichloromethane (5 mL) were placed in a reaction flask. Trifluoroacetic anhydride (TFAA, 45.0 mg, 0.3 mmol, 1.0 equiv.) was added dropwise with stirring. After stirring for 5 minutes at room temperature, coronene (90.1 mg, 0.3 mmol, 1.0 equiv.) was added, followed by the slow addition of trifluoromethanesulfonic acid (TFOH, 126.0 mg, 0.6 mmol, 2.0 equiv.). The reaction mixture was stirred at room temperature for 3 hours. The obtained crude product condensed by rotary evaporation and then purified by column chromatography over silica gel using the petroleum ether/dichloromethane (1/3, v/v) as eluent. The yellow solid product **1** was obtained with an isolation yield of 73% (114.3 mg). ^{1}H NMR (400 MHz, Chloroform-*d*) δ 9.03 (d, *J* = 8.7 Hz, 1H), 8.95 (s, 1H), 8.89 – 8.81 (m, 9H), 8.05 (d, *J* = 9.0 Hz, 2H), 6.97 (d, *J* = 8.9 Hz, 2H), 4.13 (q, *J* = 7.0 Hz, 2H), 1.46 (t, *J* = 7.0 Hz,

3H). FT-IR (KBr, cm^{-1}): 3050.5, 2979.9, 2931.3, 1900.3, 1722.6, 1639.8, 1599.5, 1571.0, 1502.3, 1473.1, 1453.5, 1421.8, 1397.0, 1346.1, 1305.6, 1273.1, 1255.5, 1220.0, 1174.1, 1166.3, 1140.7, 1114.4, 1072.5, 1041.3, 972.8, 923.4, 904.3,847.5, 812.9, 793.1, 777.3, 766.8, 704.6, 651.2, 626.2, 601.4, 567.4, 547.3, 510.3, 444.5, 420.9, 402.3. HRMS (positive ESI) m/z found (calcd for $C_{33}H_{20}O_2$+H^+): 449.1520 (449.1463). Due to the extremely low solubility of compound **1** in common solvents, we didn't obtain satisfactory ^{13}C NMR spectra.

Synthesis of coronen-1-yl(3-ethoxyphenyl) methanone (Compound 2)

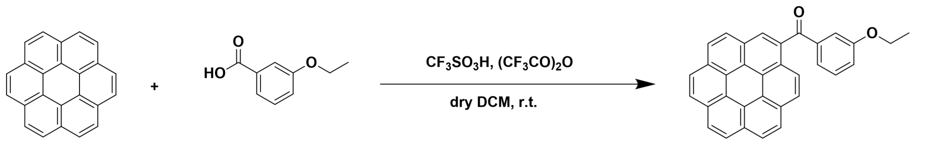

Coronene 2

3-Ethoxybenzoic acid (66.5 mg, 0.4 mmol, 1.3 equiv.) and anhydrous dichloromethane (5 mL) were placed in a reaction flask. TFAA (45.0 mg, 0.3 mmol, 1.0 equiv.) was added dropwise with stirring. After stirring for 5 minutes at room temperature, coronene (90.1 mg, 0.3 mmol, 1.0 equiv.) was added, followed by the slow addition of TFOH (126.0 mg, 0.6 mmol, 2.0 equiv.). The reaction mixture was stirred at room temperature for 3 hours. The obtained crude product condensed by rotary evaporation and then purified by column chromatography over silica gel using the petroleum ether/dichloromethane (1/4, v/v) as eluent. The yellow solid product **2** was obtained with an isolation yield of 62% (97.1 mg).^{1}H NMR (400 MHz, Chloroform-*d*) δ 9.11 (d, J = 8.7 Hz, 1H), 8.98 (s, 1H), 8.86 (dq, J = 6.2, 3.5, 2.8 Hz, 9H), 7.69 (d, J = 2.2 Hz, 1H), 7.55 (d, J = 7.8 Hz, 1H), 7.40 (t, J = 7.9 Hz, 1H), 7.22 (dd, J = 9.1, 2.7 Hz, 1H), 4.10 (q, J = 7.0 Hz, 2H), 1.41 (t, J = 7.0 Hz, 3H). FT-IR (KBr, cm^{-1}): 3049.7, 2977.3, 2932.1, 2891.6, 1907.4, 1792.1, 1655.3, 1591.1, 1532.8, 1503.1, 1484.3, 1473.4, 1452.3, 1433.5, 1388.0, 1347.4, 1324.8, 1305.0, 1271.5, 1244.9, 1207.4, 1179.3, 1167.5, 1156.0, 1139.8, 1114.8, 1081.1,1045.5, 987.6, 949.3, 920.4, 898.6, 867.5, 849.3, 826.3, 813.8, 803.4, 793.6, 771.6, 759.3, 708.6, 684.8, 658.3, 630.7, 615.6, 591.4, 546.6, 506.1, 482.8, 458.2, 421.3. HRMS (positive ESI) m/z found (calcd for $C_{33}H_{20}O_2$+H^+): 449.1552 (449.1463). Due to the extremely low solubility of compound **2** in common solvents, we didn't obtain satisfactory ^{13}C NMR spectra.

Synthesis of coronen-1-yl(2-ethoxyphenyl) methanone (Compound 3)

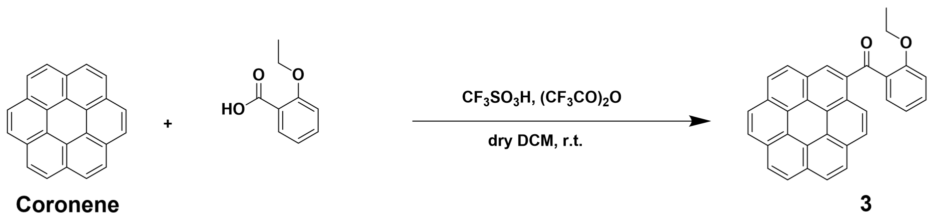

Coronene 3

2-Ethoxybenzoic acid (66.5 mg, 0.4 mmol, 1.3 equiv.) and anhydrous dichloromethane (5 mL) were placed in a reaction flask. TFAA (45.0 mg, 0.3 mmol, 1.0 equiv.) was added dropwise with stirring. After stirring for 5 minutes at room temperature, coronene (90.1 mg, 0.3 mmol, 1.0 equiv.) was added, followed by the slow addition of TFOH (126.0 mg, 0.6 mmol, 2.0 equiv.). The reaction mixture was stirred at room temperature for 3 hours. The obtained crude product condensed by rotary evaporation and then purified by column chromatography over silica gel using the

petroleum ether/dichloromethane (1/2, v/v) as eluent. The yellow solid product **3** was obtained with an isolation yield of 57% (89.3 mg). ^{1}H NMR (400 MHz, Chloroform-*d*) δ 9.48 (d, J = 8.8 Hz, 1H), 8.98 – 8.80 (m, 10H), 8.02 – 7.94 (m, 1H), 7.60 (t, J = 7.8 Hz, 1H), 7.21 (t, J = 7.6 Hz, 1H), 6.97 (d, J = 8.4 Hz, 1H), 3.63 (q, J = 6.8 Hz, 2H), 0.17 (t, J = 7.0 Hz, 3H). FT-IR (KBr, cm^{-1}):3442.7, 3049.7, 2978.2, 2928.9, 2878.6, 1910.5, 1807.5, 1658.5, 1607.7, 1592.2, 1532.9, 1500.9, 1482.2, 1468.4, 1453.3, 1390.6, 1345.7, 1325.6, 1304.2, 1291.4, 1259.0, 1248.2, 1216.0, 1183.0, 1158.7, 1141.2, 1122.3, 1075.3, 1040.1, 973.5, 929.7, 906.5, 849.8, 836.6, 814.3, 799.6, 783.1, 761.6, 668.4, 655.2, 644.4, 630.5, 600.2, 550.9, 512.2, 472.3, 419.9. HRMS (positive ESI) m/z found (calcd for $C_{33}H_{20}O_2+H^+$): 449.1551 (449.1463). Due to the extremely low solubility of compound **3** in common solvents, we didn't obtain satisfactory ^{13}C NMR spectra.

Synthesis of deuterated compound 1

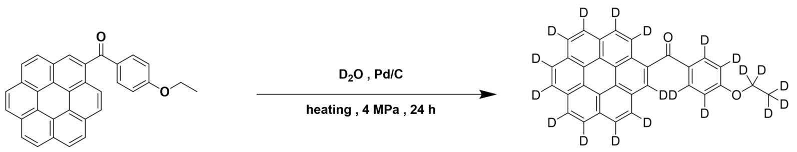

1

230 °C, 300 mg Pd/C → **1^{D1}** 46 atom% D, yield 69%

240 °C, 800 mg Pd/C → **1^{D2}** 75 atom% D, yield 23%

Into a 0.13 L Teflon-lined autoclave were added 200 mg undeuterated compound **1**, 30 mL D_2O and 300 mg Pd/C (10 wt % Pd on activated carbon). The reaction mixture was heated to 230 °C and 4 MPa for 24 h. The deuteration reaction was performed in a safe laboratory in our institute. After deuteration, the autoclave was cooled to room temperature. The reaction mixture was then extracted with dichloromethane. The obtained organic solution was dried over anhydrous sodium sulphate, condensed by rotary evaporation, and then purified by column chromatography over silica gel using the petroleum ether/dichloromethane (1:1) as eluent. The yellow solid compound **1^{D1}** was obtained with an isolation yield of 69% (153 mg). The deuteration yield of compound **1^{D1}** was determined by ^{1}H NMR to be 46% using 1,4-dioxane as reference.

To increase the deuteration yield of compound **1**, the loading of Pd/C was increased to 800 mg, and the temperature was raised to 240 °C. The other reaction conditions are the same. The resultant compound **1^{D2}** has a deuteration yield of 75% with isolation yeld of 23% (44.2 mg).

Preparation of afterglow materials by doping compounds 1-3 into organic matrices

For the preparation of **1**-DMI-0.1% materials, 200 μL compound **1** in dichloromethane (1 mg/mL) and 200 mg dimethyl isophthalate (DMI) were added into a 3 mL sample bottle and then heated to 80 °C to give a molten mixture. Subsequently, the sample bottle was transferred to a bath of liquid nitrogen to immediately solidify the molten mixture. After standing at room temperature for tens of minutes, **1**-DMI-0.1% sample was obtained. Other samples at different doping concentrations or using different compounds and different organic matrices were prepared through similar processes.

Table S1. Selected examples of organic afterglow materials with corresponding lifetimes and afterglow efficiency under ambient conditions.

Chemical structure	τ / ms	Φ_{AG}	Φ_P	Brightness	$L(t)$ t = 1 s	Reference
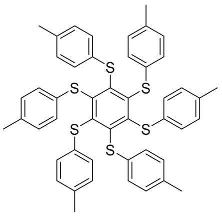	0.003	-	100%	-	-	*Dyes Pigm.* **2014**, 110, 113.
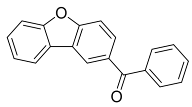	232	34.5%	-	-	-	*Chem* **2016**, 1, 592.
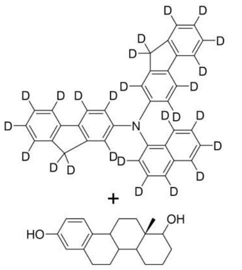	1000	-	50%	-	-	*Adv. Mater.* **2020**, 32, 2001348.
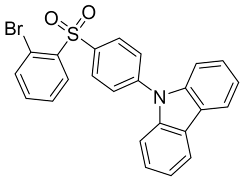	180	-	52.1%	-	352	*Angew. Chem. Int. Ed.* **2020**, 59, 17451.
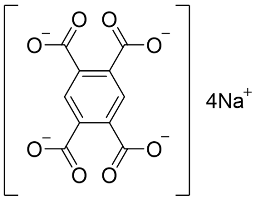	169	-	66.9%	-	-	*Nat. Mater.* **2021**, 1539–1544.
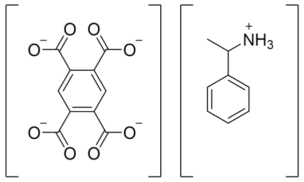	185		96.5%	-	-	*Nat. Mater.* **2021**, 1539–1544.

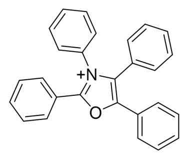	710	-	36.5%	-	-	*Nat. Commun.* **2018**, 9, 2963.
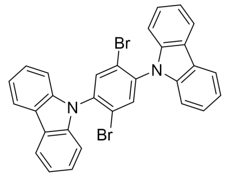	560	-	38.1%	-	-	*J. Phys. Chem. Lett.* **2019**, 10, 595.
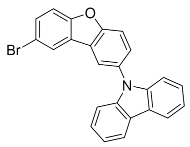	540	-	41.2%	-	-	*Nat. Commun*. **2019**, 10, 1595.
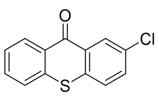	1.3	74.7%	-	-	-	*J. Mater. Chem. C.* **2019**, 7, 12502.
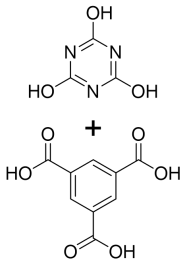	1670	46.1%	-	-	-	*Nat. Commun*. **2020**, 11, 4802.
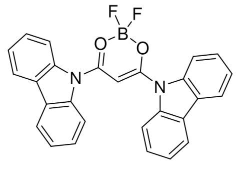	240	44.9%	-	-	-	*Nat. Commun*. **2020**, 11, 842.
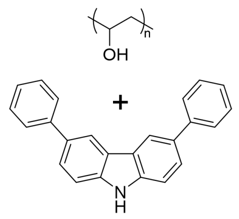	2045	-	0.81%	0.1584 $cd{\cdot}m^{-2}$	-	*J. Am. Chem. Soc.* **2021**, 143, 13675-13685.

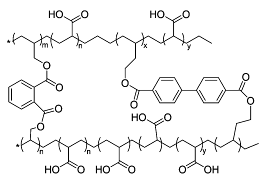	1200	-	37.5%	-	-	*Nat. Commun.* **2020**, 11, 944.
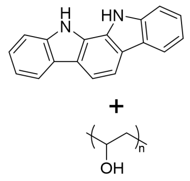	2040	-	44.1%	-	-	*Adv. Funct. Mater.* **2022**, 2208895.
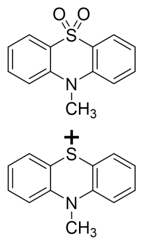	afterglow duration 25 min	-	43%	-	-	*Adv. Mater.* **2021**, 33, 2007811.
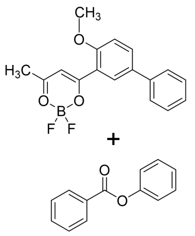	300	70%	-	-	1373	*Angew. Chem. Int. Ed,* **2021**, 60, 17138.
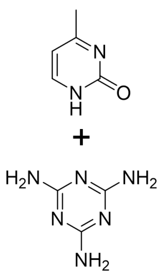	336.8	37.1%	-	-	-	*Adv. Opt. Mater.* **2022**, 10, 2200451.
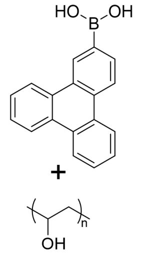	3290	33.1%	-	-	-	*Adv. Mater.* **2022**, 34, 2108333.
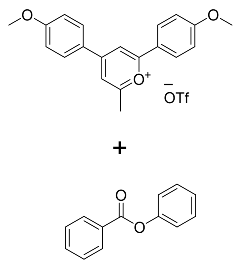	225	48%	-	-	-	*Chem. Eng. J.* **2022**, 431, 134197.

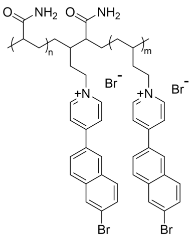	10	67.1%	-	-	-	*Angew. Chem. Int. Ed.* **2022**, 61, e202213097.
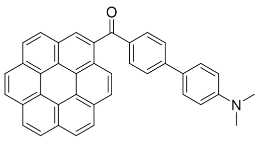	1500	-	38%	60 cd·m^{-2}	15510	*Angew. Chem. Int. Ed.* **2025**, 64, e202513685.
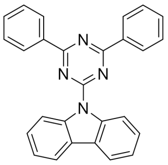	1320	-	25.3%	0.1340 cd·m^{-2}	-	*Nat. Mater.* **2015**, 14, 685-690.
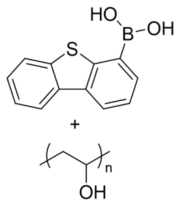	1060	-	1.3%	81 lm·m^{-2}	-	*Adv. Opt. Mater.* **2023**, 12, 2302424.
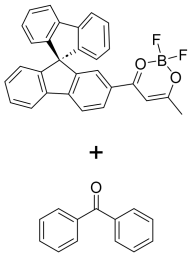	600	30%	-	8 lm·m^{-2}	-	*Adv. Opt. Mater.* **2021**, 9, 2100353.
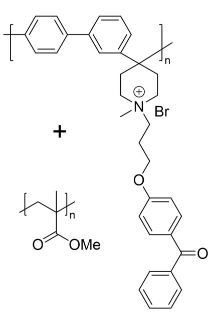	1258	-	2.7%	0.0099 cd·m^{-2}	-	*Adv. Mater.* **2022**, 34, 2204415.
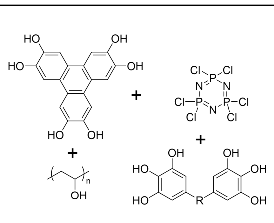	1755	-	6.8%	0.2231 cd·m^{-2}	-	*J. Am. Chem. Soc.* **2022**, 144, 6107-6117.

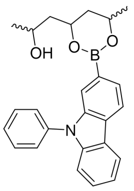	4440	-	5.1%	69.7 lm·m^{-2}	-	*Chem. Sci.* **2023**, 14, 5177-5181.
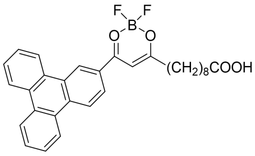	1970		20.8%	-	4219	*Adv. Opt. Mater*. **2024**, 12, 2302311.
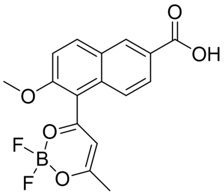	1300	0.47%	-	-	3898	*ACS Applied Materials & Interfaces*. **2022**, 14, 1587-1600.
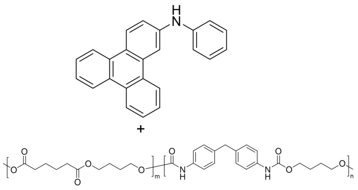	725	-	12.8%	0.611 cd·m^{-2}	-	*Adv. Mater.* **2024**, 36, 2409642.
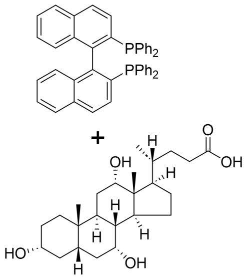	560	-	5.7%	49.4 cd·m^{-2}	1816	*Adv. Mater.* **2025**, 37, 2500953.
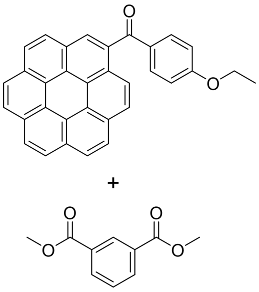	3190	-	17.5%	73 cd·m^{-2}	16372	This work

Table S2. Photophysical properties of the coronene derivatives in dichloromethane (DCM), toluene (PhMe) and ethyl acetate (EA): absorption peaks (λ_{abs}), molar absorption coefficients (ε), and fluorescence maxima (λ_F) (excited at 365 nm).

	DCM				PhMe				EA			
	λ_{abs} (nm)	ε ($10^5 \cdot M^{-1} \cdot cm^{-1}$)	λ_F (nm)	Φ %	λ_{abs} (nm)	ε ($10^5 \cdot M^{-1} \cdot cm^{-1}$)	λ_F (nm)	Φ %	λ_{abs} (nm)	ε ($10^5 \cdot M^{-1} \cdot cm^{-1}$)	λ_F (nm)	Φ %
**1**	307	1.31	442	1.69	308	1.32	442	0.95	305	1.28	439	1.18
	344	0.34			345	0.34			343	0.31		
**2**	305	1.17	446	1.72	307	1.28	446	6.34	304	1.35	443	3.06
	344	0.29			345	0.36			342	0.33		
**3**	305	1.23	451	1.59	306	1.30	448	1.21	304	1.14	446	4.00
	343	0.33			344	0.36			342	0.29		

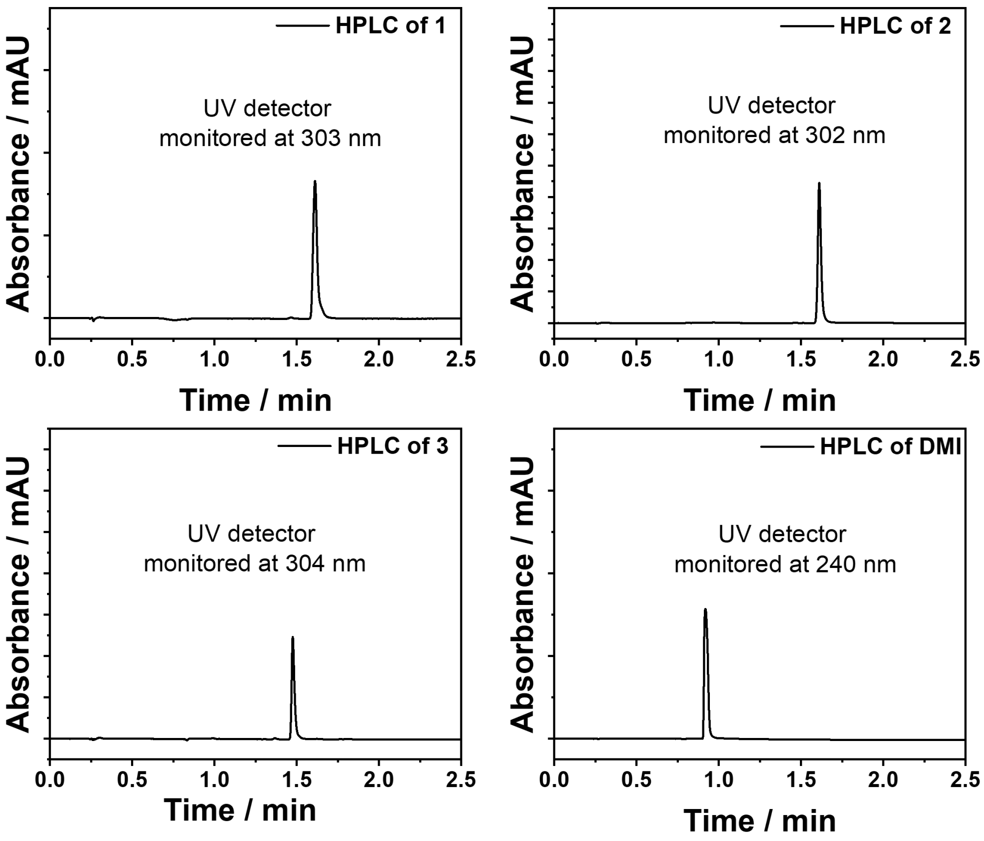

Figure S1. HPLC profile of compound **1-3** and DMI.

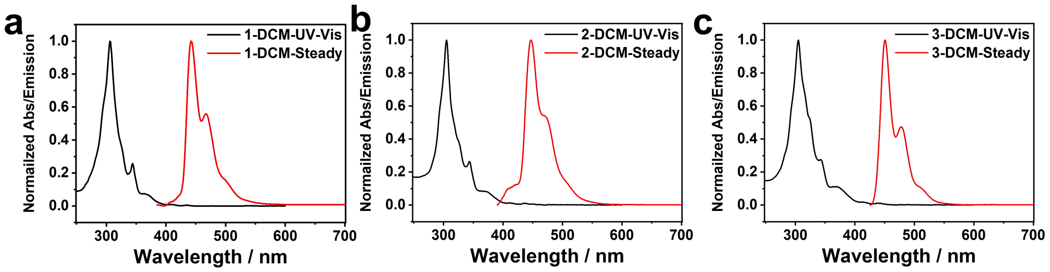

Figure S2. UV-Vis absorption spectra (normalized) and the steady-state emission spectra (excited at 365 nm) of coronene derivatives in dichloromethane (DCM) solution at room temperature.

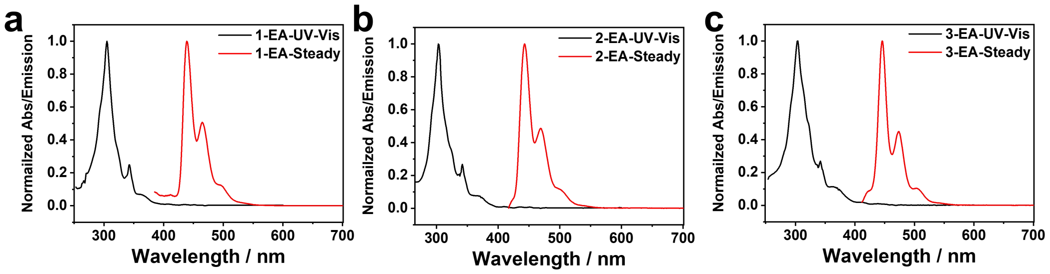

Figure S3. UV-Vis absorption spectra (normalized) and the steady-state emission spectra (excited at 365 nm) of coronene derivatives in EA solution at room temperature.

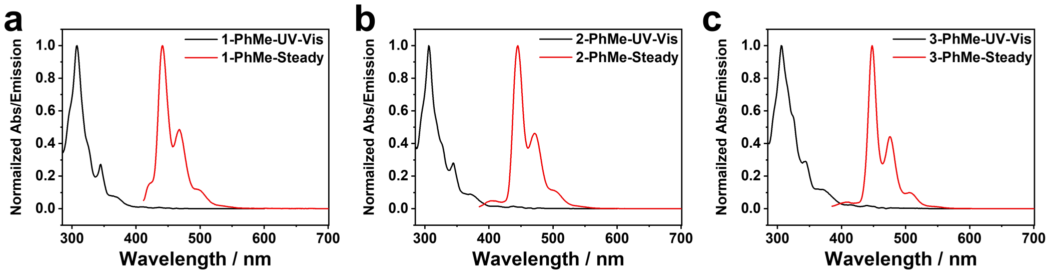

Figure S4. UV-Vis absorption spectra (normalized) and the steady-state emission spectra (excited at 365 nm) of coronene derivatives in toluene (PhMe) solution at room temperature.

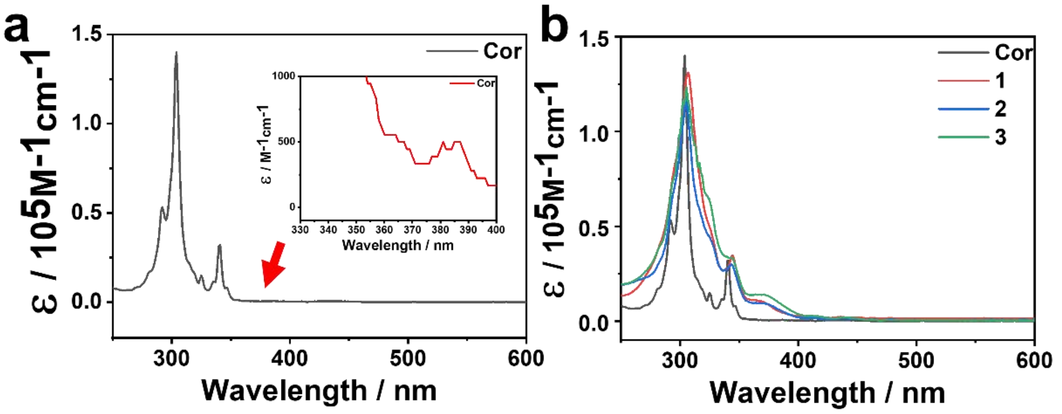

Figure S5. (**a**) UV-Vis spectrum of coronene in dichloromethane. Inset shows the weak absorption in the range of 350-400 nm. (**b**) UV-vis spectra of coronene and **CoDe**. Coronene exhibits D_{6h} symmetry. The S_0-S_1 and S_0-S_2 transitions are symmetry-forbidden in the coronene system with oscillator strength (f_{osc}) close to zero as revealed by TD-B3LYP/G-def2-TZVP(-f) calculation (*Adv. Mater.* **2025**, *37*, e18750), while the S_0-S_3 transition is both spin- and symmetry-allowed and thus exhibits significantly higher f_{osc} than S_0-S_1 and S_0-S_2 transitions; the TD-DFT calculations only consider electronic structures but don't consider the contribution of vibration to S_0-S_n transitions. According to the reported studies (*J. Chem. Phys.* **2017**, *146*, 044309; *J. Phys. Chem. Lett.* **2017**, *8*, 3683), it is known that the e_{2g} vibrational levels in the S_1 state can borrow intensity from S_3 state in coronene system, which is the reason for its absorption at around 385 nm as shown in Figure S5a.

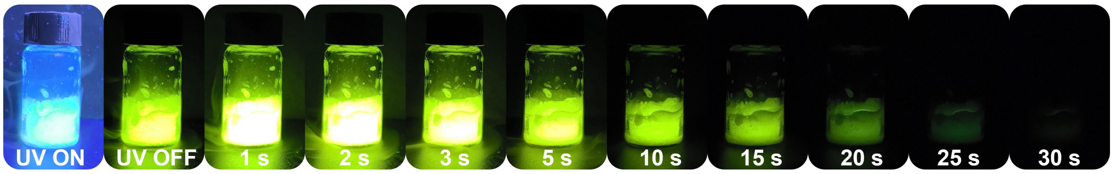

Figure S6. Afterglow photograph of **1**-EA solution at 77 K.

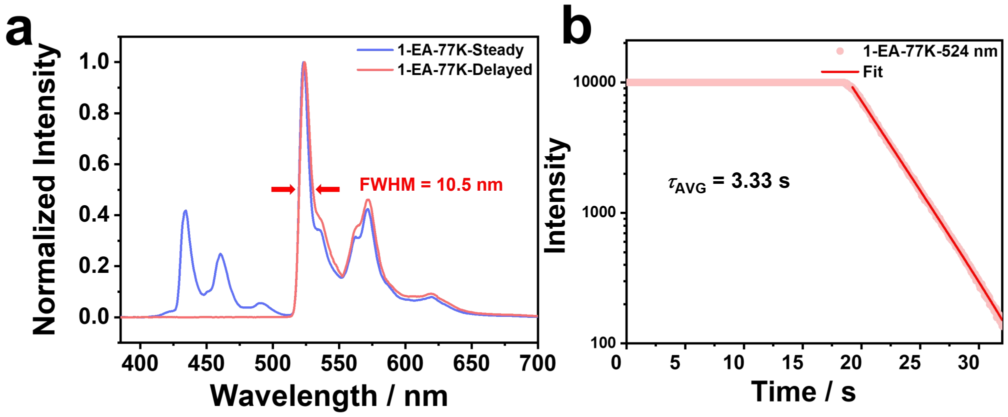

Figure S7. (**a**) Steady-state and delayed emission (1 ms delay) spectra and (**b**) phosphorescence decay of compound **1** in EA solution at 77 K (excited at 365 nm).

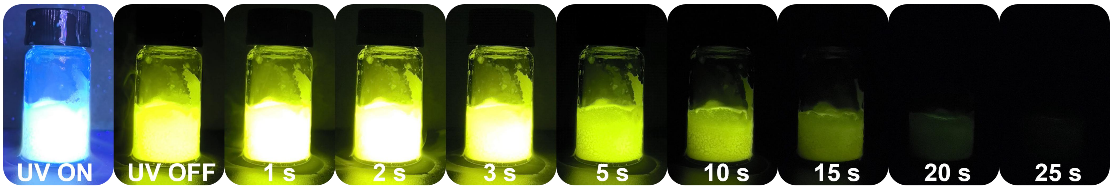

Figure S8. Afterglow photograph of **2**-EA solution at 77 K.

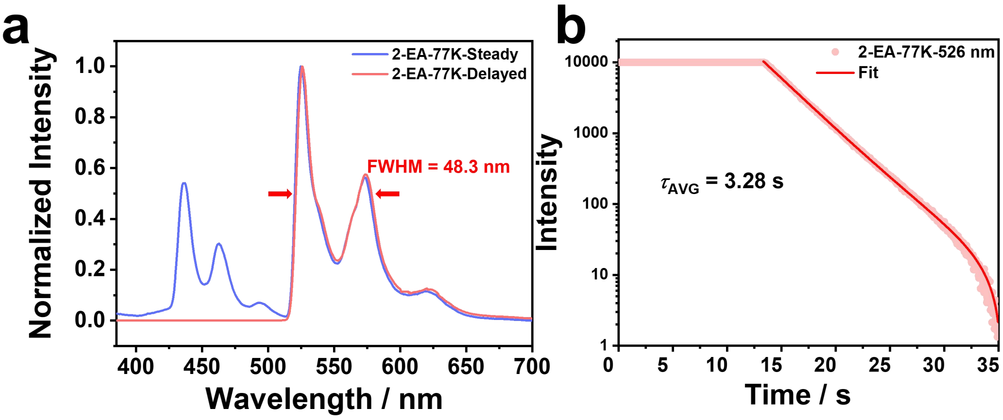

Figure S9. (**a**) Steady-state and delayed emission (1 ms delay) spectra and (**b**) phosphorescence decay of compound **2** in EA solution at 77 K (excited at 365 nm).

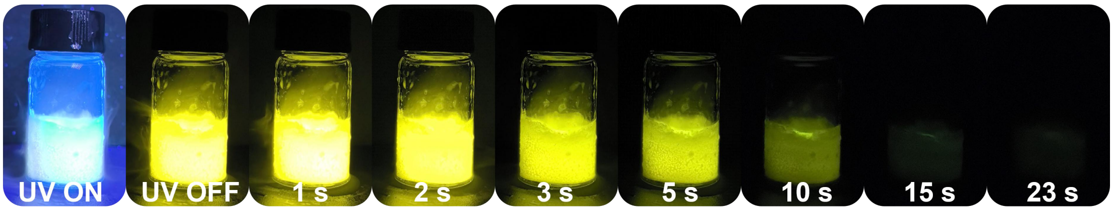

Figure S10. Afterglow photograph of **3**-EA solution at 77 K.

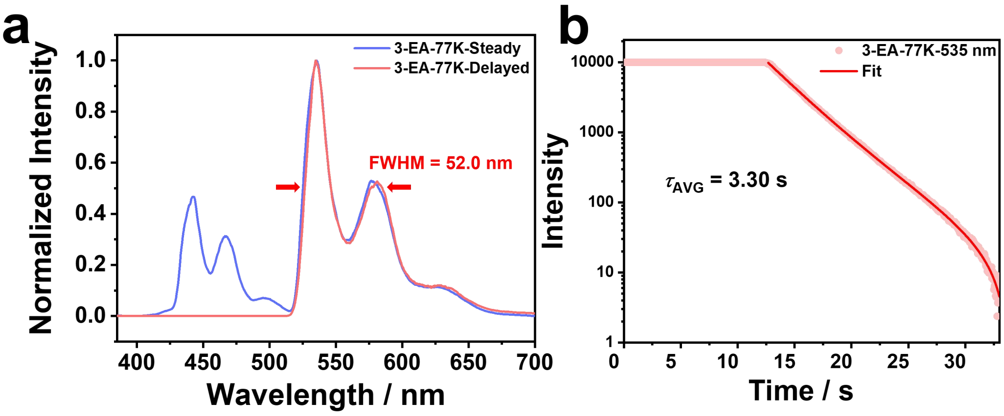

Figure S11. (**a**) Steady-state and delayed emission (1 ms delay) spectra and (**b**) phosphorescence decay of compound **3** in EA solution at 77 K (excited at 365 nm).

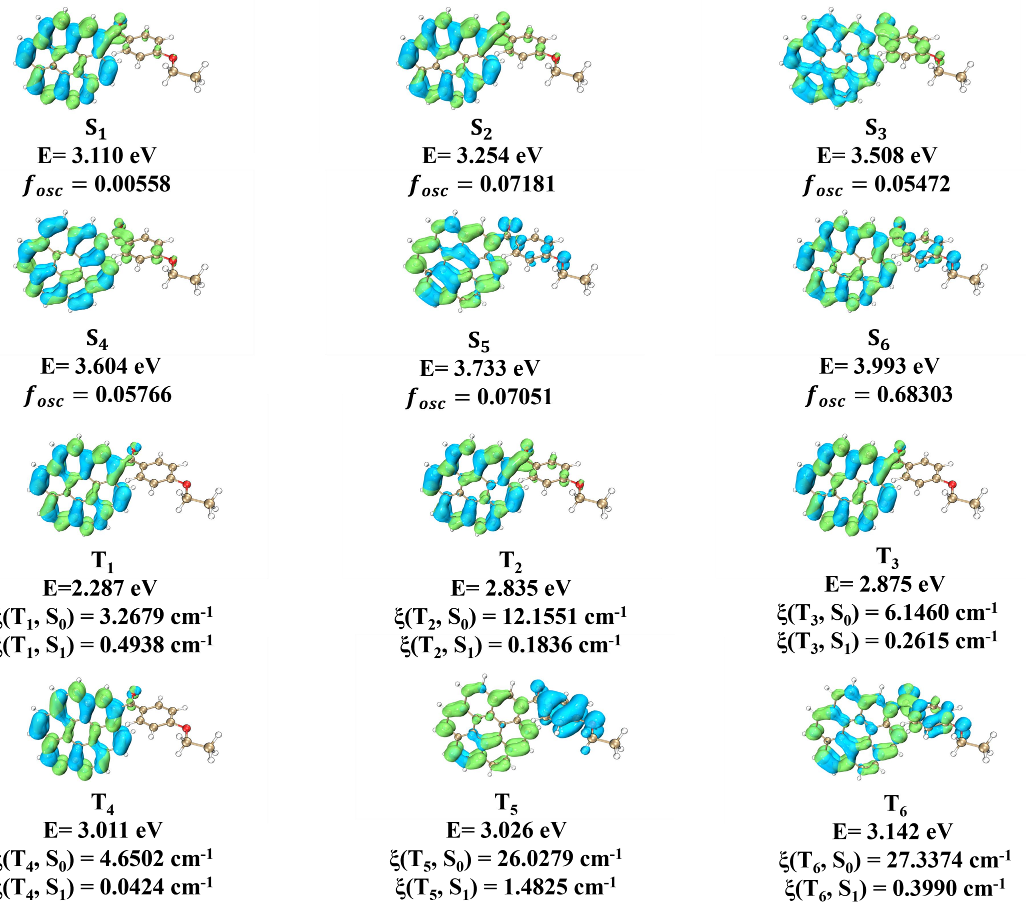

Figure S12. Iso-surface maps of electron-hole density difference of **1**'s S_n and T_n states, where blue and green iso-surfaces correspond to hole and electron distributions, respectively, and SOCME values. The ground-state geometry was optimized by a DFT calculation using B3LYP functional and 6-31g(d,p) basis set. The singlet excited states and triplet excited states were calculated on Gaussian 16 program (Revision A.03) with B3LYP functional and 6-31g(d,p) functional. Spin-orbit coupling (SOC) matrix elements between the singlet excited states and triplet excited states were calculated with spin-orbit mean-field (SOMF) methods on ORCA 5.0.3 program with B3LYP functional and 6-31g(d,p) basis set.

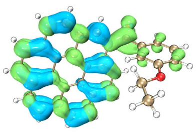

S_1
E= 3.086 eV
f_{osc} = 0.00688

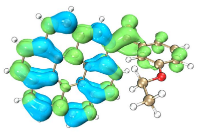

S_2
E= 3.197 eV
f_{osc} = 0.06569

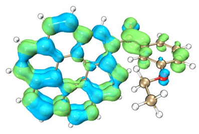

S_3
E= 3.451 eV
f_{osc} = 0.01248

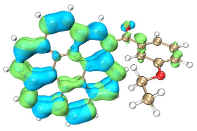

S_4
E= 3.517 eV
f_{osc} = 0.03929

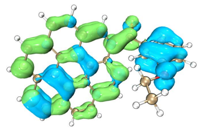

S_5
E= 3.635 eV (341.13 nm)
f_{osc} = 0.05766

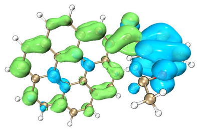

S_6
E= 3.757 eV
f_{osc} = 0.01785

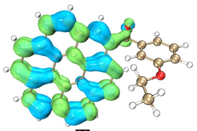

T_1
E= 2.279 eV
$\xi(T_1, S_0) = 4.2128\ cm^{-1}$
$\xi(T_1, S_1) = 0.6641\ cm^{-1}$

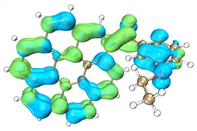

T_2
E= 2.780 eV
$\xi(T_2, S_0) = 21.6689\ cm^{-1}$
$\xi(T_2, S_1) = 0.7457\ cm^{-1}$

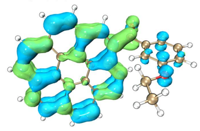

T_3
E= 2.845 eV
$\xi(T_3, S_0) = 9.4612\ cm^{-1}$
$\xi(T_3, S_1) = 0.8105\ cm^{-1}$

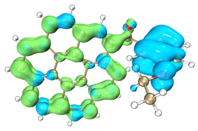

T_4
E= 2.902 eV
$\xi(T_4, S_0) = 11.5701\ cm^{-1}$
$\xi(T_4, S_1) = 1.2001\ cm^{-1}$

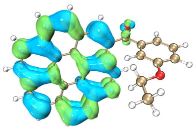

T_5
E= 3.011 eV
$\xi(T_5, S_0) = 3.6832\ cm^{-1}$
$\xi(T_5, S_1) = 0.2670\ cm^{-1}$

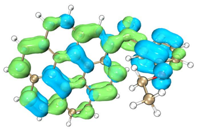

T_6
E= 3.089 eV
$\xi(T_6, S_0) = 32.7164\ cm^{-1}$
$\xi(T_6, S_1) = 0.6687\ cm^{-1}$

Figure S13. Iso-surface maps of electron-hole density difference of **2**'s S_n and T_n states, where blue and green iso-surfaces correspond to hole and electron distributions, respectively, and SOCME values. The ground-state geometry was optimized by a DFT calculation using B3LYP functional and 6-31g(d,p) basis set. The singlet excited states and triplet excited states were calculated on Gaussian 16 program (Revision A.03) with B3LYP functional and 6-31g(d,p) functional. Spin-orbit coupling (SOC) matrix elements between the singlet excited states and triplet excited states were calculated with spin-orbit mean-field (SOMF) methods on ORCA 5.0.3 program with B3LYP functional and 6-31g(d,p) basis set.

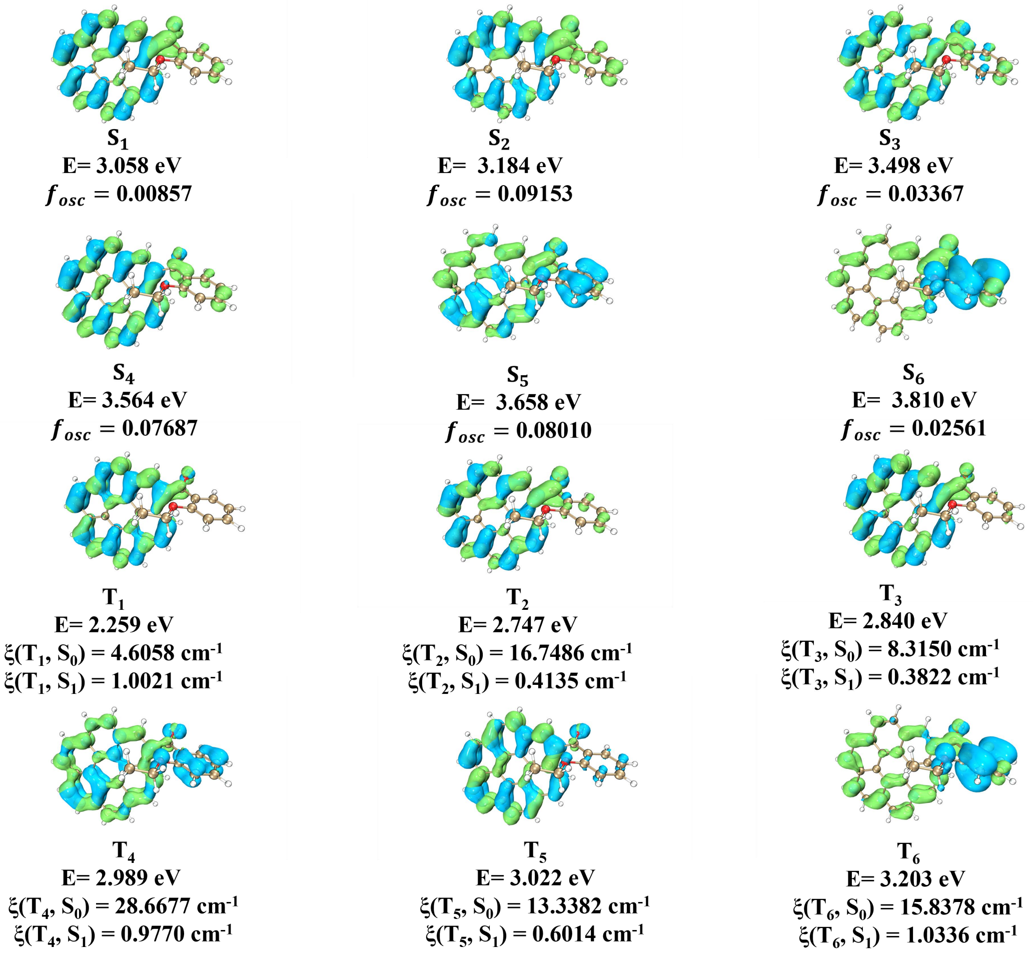

Figure S14. Iso-surface maps of electron-hole density difference of **3**'s S_n and T_n states, where blue and green iso-surfaces correspond to hole and electron distributions, respectively, and SOCME values. The ground-state geometry was optimized by a DFT calculation using B3LYP functional and 6-31g(d,p) basis set. The singlet excited states and triplet excited states were calculated on Gaussian 16 program (Revision A.03) with B3LYP functional and 6-31g(d,p) functional. Spin-orbit coupling (SOC) matrix elements between the singlet excited states and triplet excited states were calculated with spin-orbit mean-field (SOMF) methods on ORCA 5.0.3 program with B3LYP functional and 6-31g(d,p) basis set.

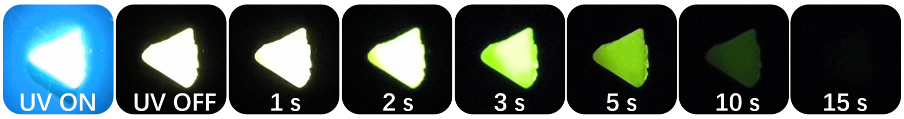

Figure S15. Photograph of **1**-PhB-0.1% material afterglow object.

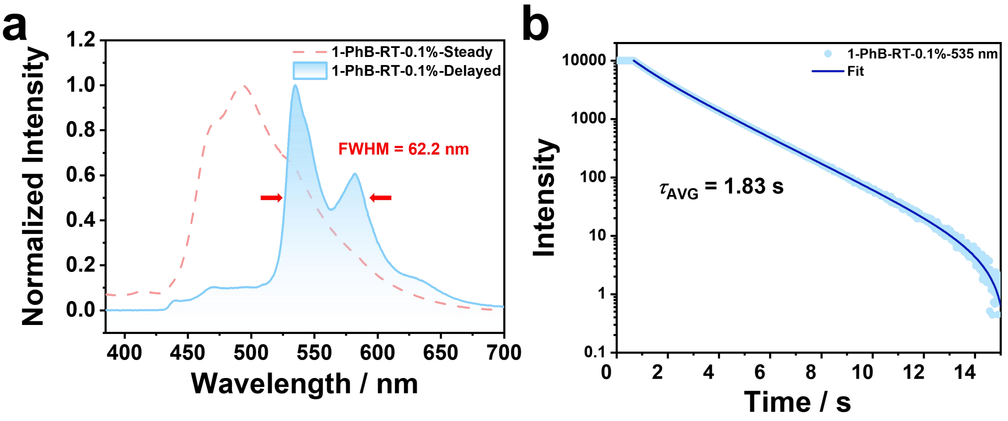

Figure S16. (**a**) Steady-state and delayed emission (1 ms delay) spectra and (**b**) phosphorescence decay of **1**-PhB-0.1% samples at room temperature (excited at 365 nm).

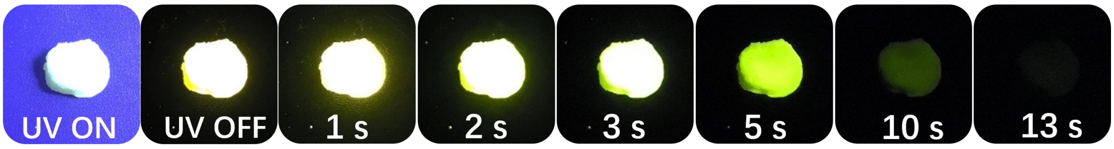

Figure S17. Photograph of **1**-MeOBP-0.1% material afterglow object.

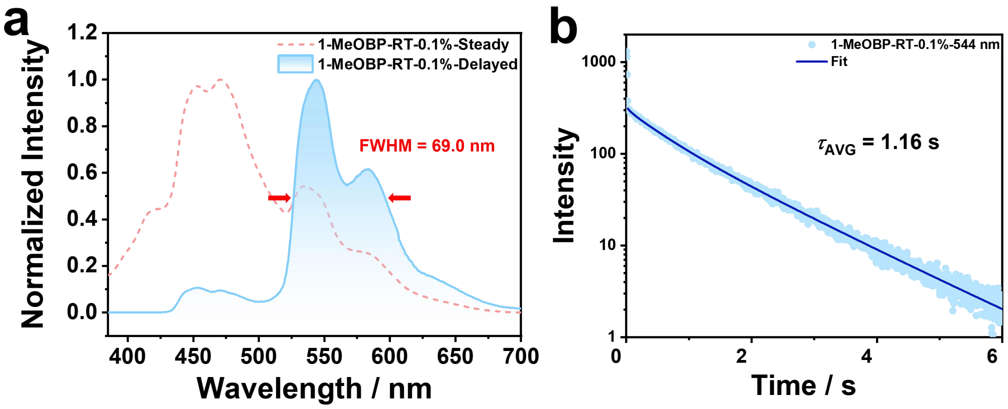

Figure S18. (**a**) Steady-state and delayed emission (1 ms delay) spectra and (**b**) phosphorescence decay of **1**-MeOBP-0.1% samples at room temperature (excited at 365 nm).

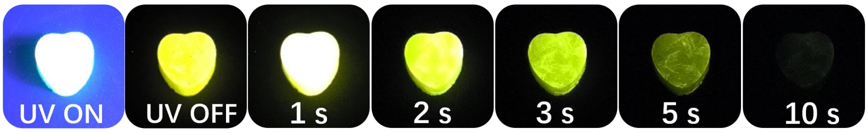

Figure S19. Photograph of **1**-MeOPhB-0.1% material afterglow object.

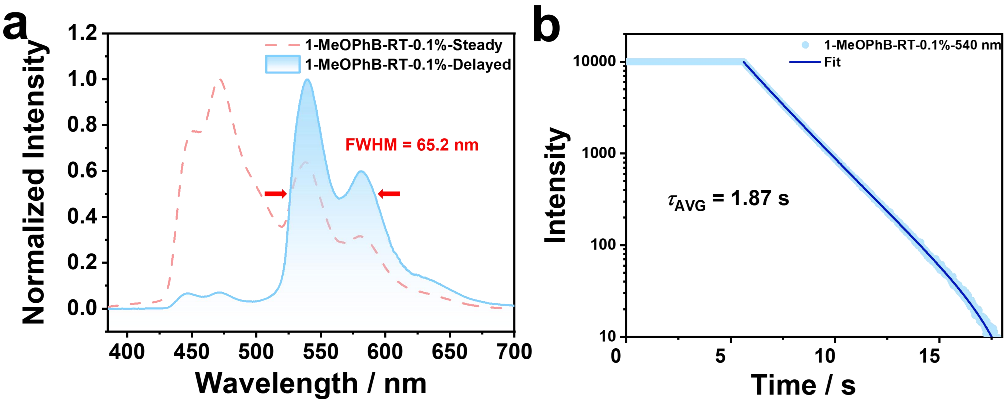

Figure S20. (**a**) Steady-state and delayed emission (1 ms delay) spectra and (**b**) phosphorescence decay of **1**-MeOPhB-0.1% samples at room temperature (excited at 365 nm).

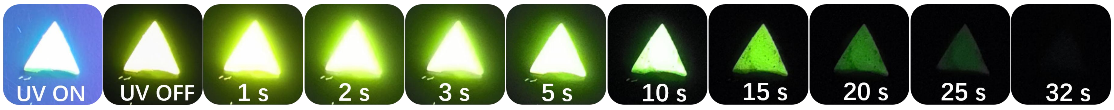

Figure S21. Photograph of **1**-DMI-0.1% material afterglow object.

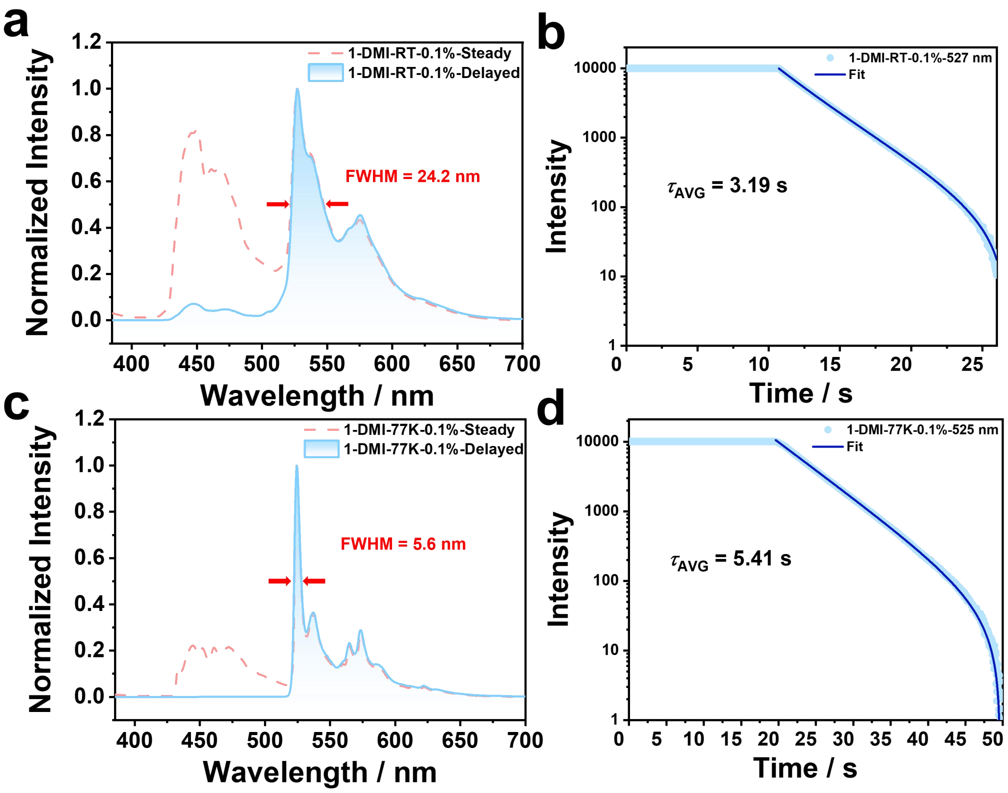

Figure S22. (**a**, **b**) Room temperature and (**c**, **d**) 77 K steady-state, delayed emission spectra (1 ms delay) and phosphorescence decay profiles of **1**-DMI-0.1% samples (excited at 365 nm).

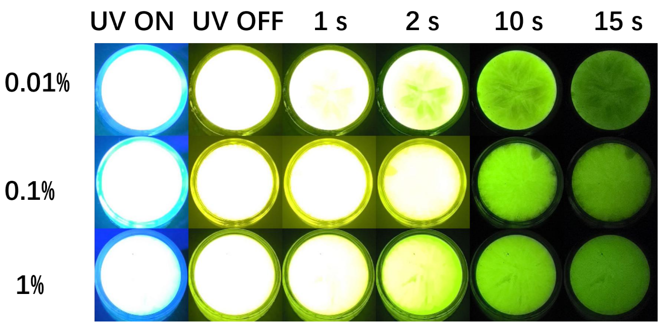

Figure S23. Photograph of **1**-DMI-(0.01%, 0.1%, 1%) samples.

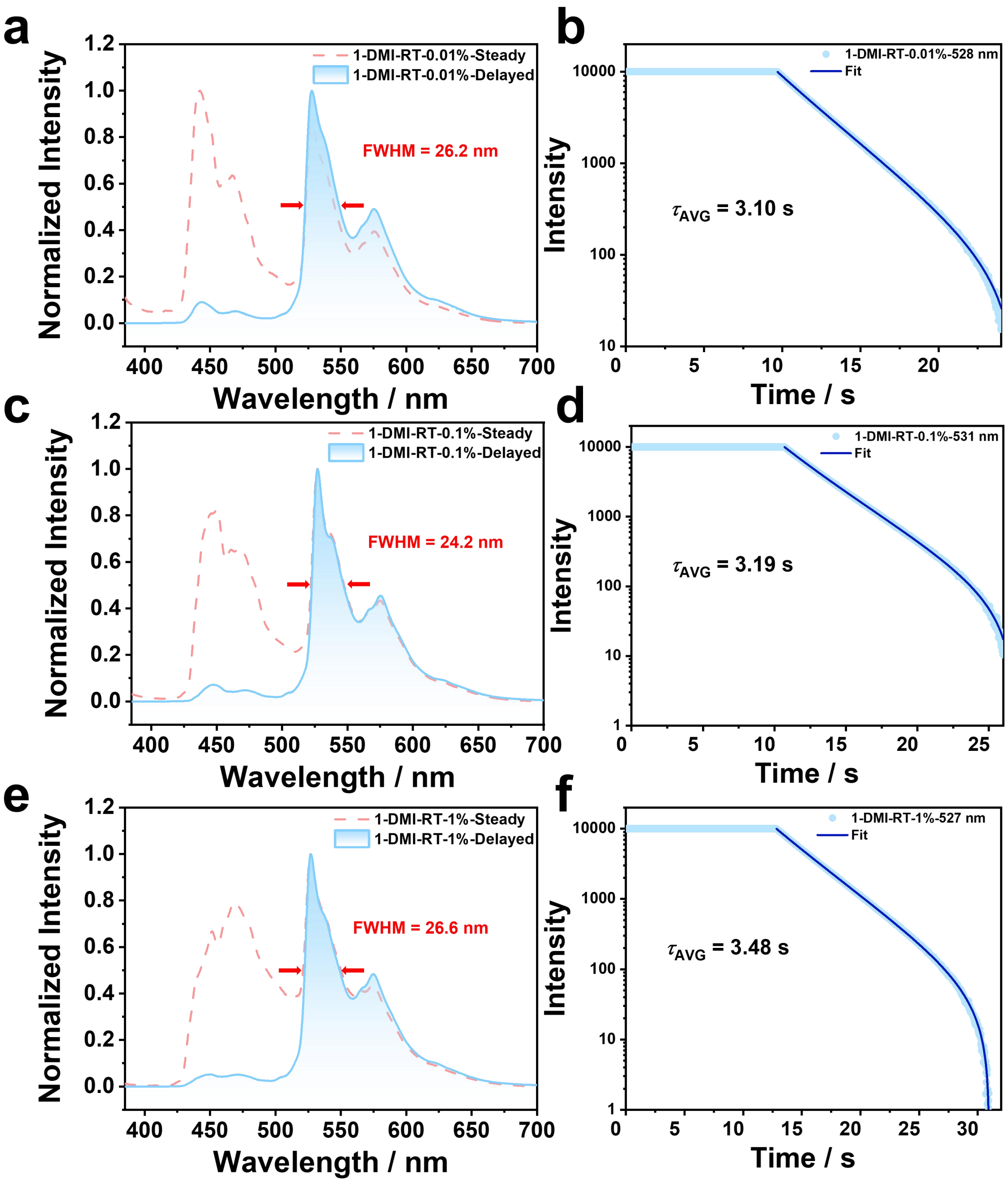

Figure S24. Room temperature steady-state, delayed emission spectra (1 ms delay) and phosphorescence decay profiles of **1**-DMI-(0.01%, 0.1%, 1%) samples (excited at 365 nm). The afterglow brightness of these samples is similar at different concentrations (Fig. S22). The

1-DMI-0.1% sample shows a smaller FWHM than 1-DMI-0.01% and 1-DMI-1%. Therefore, we choose 0.1% doping concentration in this study.

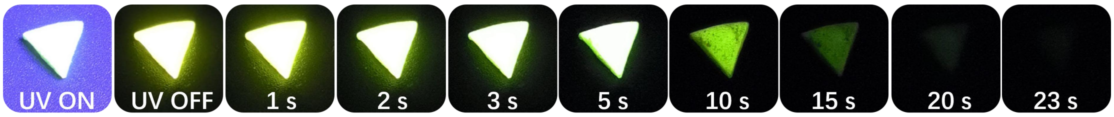

Figure S25. Photograph of **1**-DMI-0.1% material afterglow object (excited at 385 nm).

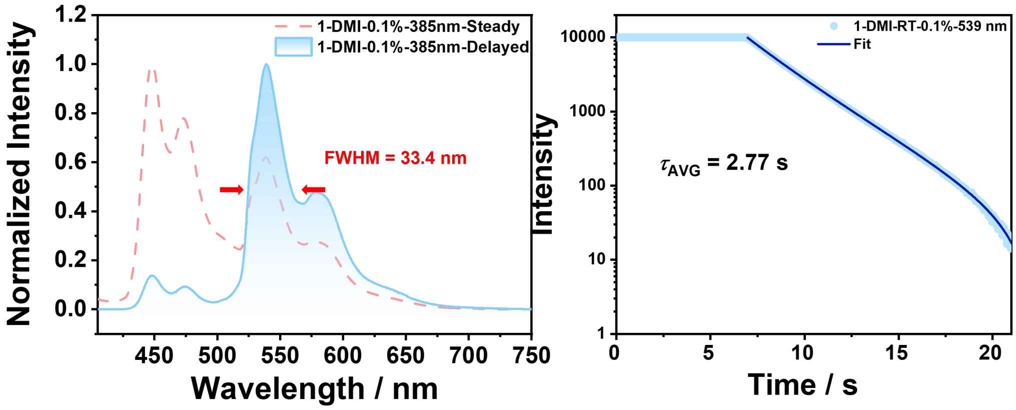

Figure S26. (a) Steady-state and delayed emission (1 ms delay) spectra and **(b)** phosphorescence decay of **1**-DMI-0.1% samples at room temperature (excited at 385 nm).

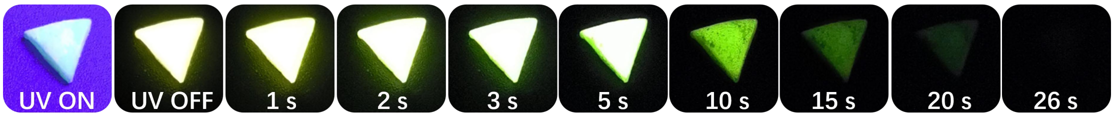

Figure S27. Photograph of **1**-DMI-0.1% material afterglow object (excited at 405 nm).

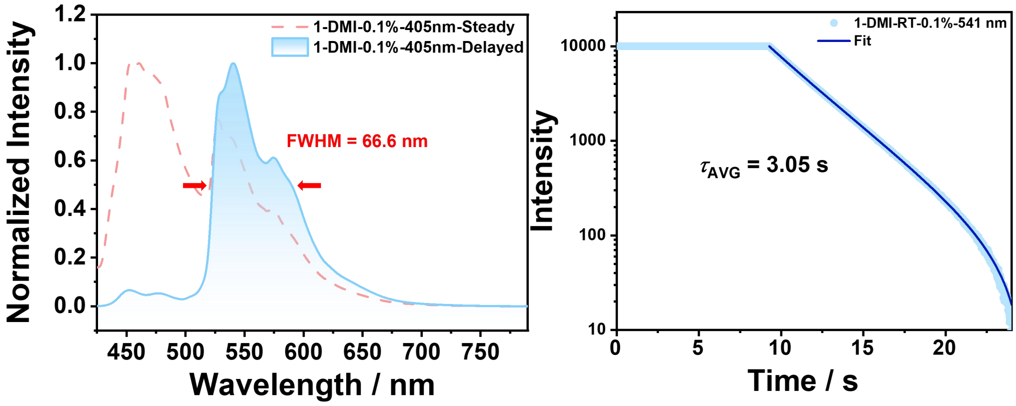

Figure S28. (a) Steady-state and delayed emission (1 ms delay) spectra and **(b)** phosphorescence decay of **1**-DMI-0.1% samples at room temperature (excited at 405 nm).

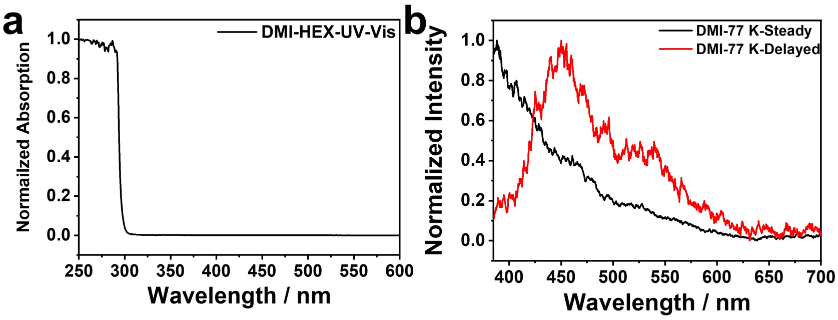

Figure S29. **(a)** Normalized UV-vis absorption spectra, **(b)** steady-state and delayed emission spectra (excited at 365 nm) of DMI in n-hexane solution at 77K.

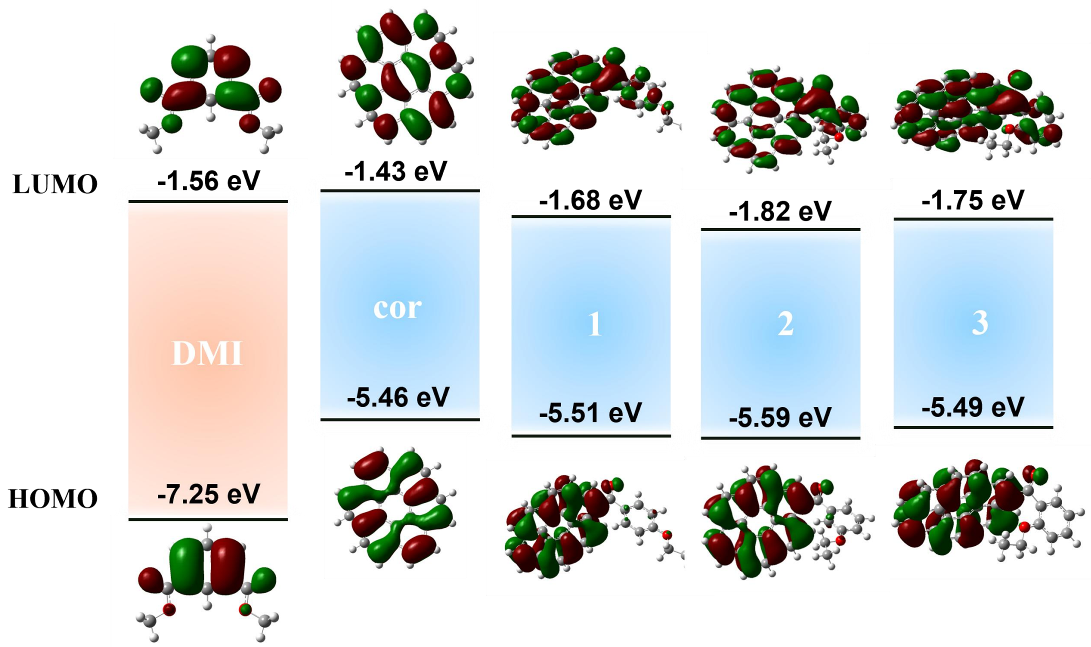

Figure S30. HOMO/LUMO energy levels of DMI, coronene and its derivatives calculated at B3LYP/6-31g(d,p).

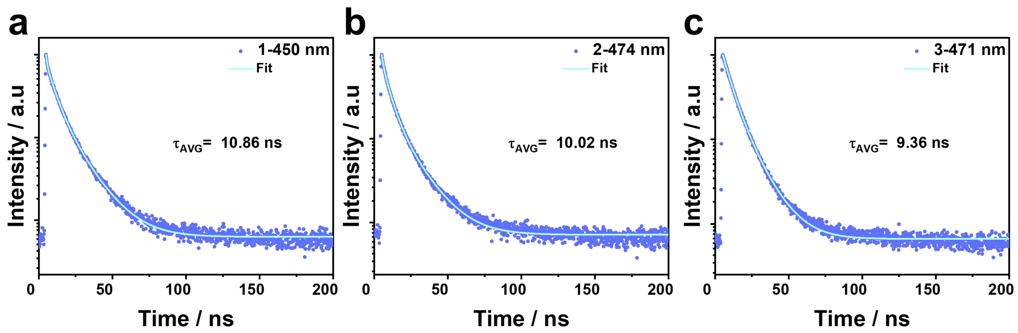

Figure S31. **(a)** The prompt fluorescence decay profiles of **1**-DMI-0.1% sample excited at 365 nm and monitored at 450 nm. **(b)** The prompt fluorescence decay profiles of **2**-DMI-0.1% sample excited at 365 nm and monitored at 474 nm. **(c)** The prompt fluorescence decay profiles of **3**-DMI-0.1% sample excited at 365 nm and monitored at 471 nm.

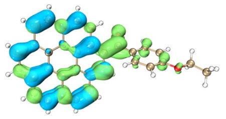

$\mathbf{S_1}$
$E = 3.063$ eV
$f_{osc} = 0.00990$

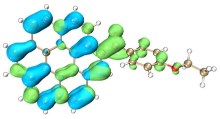

$\mathbf{S_2}$
$E = 3.201$ eV
$f_{osc} = 0.10500$

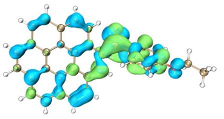

$\mathbf{S_3}$
$E = 3.541$ eV
$f_{osc} = 0.01570$

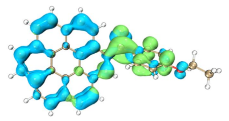

$\mathbf{S_4}$
$E = 3.598$ eV
$f_{osc} = 0.11086$

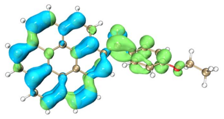

$\mathbf{S_5}$
$E = 3.627$ eV
$f_{osc} = 0.19271$

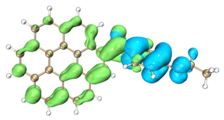

$\mathbf{S_6}$
$E = 3.984$ eV
$f_{osc} = 0.26624$

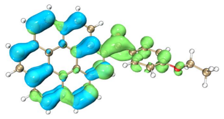

$\mathbf{T_1}$
$E = 2.275$ eV
$\xi(T_1, S_0) = 1.8027\ cm^{-1}$
$\xi(T_1, S_1) = 0.9312\ cm^{-1}$

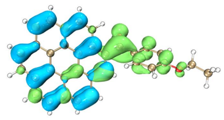

$\mathbf{T_2}$
$E = 2.781$ eV
$\xi(T_2, S_0) = 3.1239\ cm^{-1}$
$\xi(T_2, S_1) = 0.3484\ cm^{-1}$

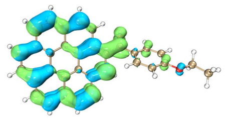

$\mathbf{T_3}$
$E = 2.836$ eV
$\xi(T_3, S_0) = 7.0039\ cm^{-1}$
$\xi(T_3, S_1) = 0.4314\ cm^{-1}$

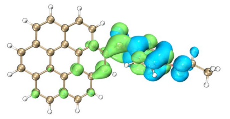

$\mathbf{T_4}$
$E = 2.965$ eV
$\xi(T_4, S_0) = 27.8804\ cm^{-1}$
$\xi(T_4, S_1) = 1.1577\ cm^{-1}$

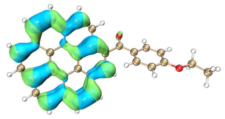

$\mathbf{T_5}$
$E = 3.019$ eV
$\xi(T_5, S_0) = 1.3814\ cm^{-1}$
$\xi(T_5, S_1) = 0.2791\ cm^{-1}$

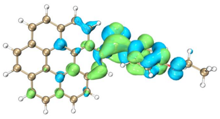

$\mathbf{T_6}$
$E = 3.214$ eV
$\xi(T_6, S_0) = 32.9706\ cm^{-1}$
$\xi(T_6, S_1) = 0.8641\ cm^{-1}$

Figure S32. Iso-surface maps of electron-hole density difference of deuterated compound **1**'s S_n and T_n states, where blue and green iso-surfaces correspond to hole and electron distributions, respectively, and SOCME values. The ground-state geometry was optimized by a DFT calculation using B3LYP functional and 6-31g(d,p) basis set. The singlet excited states and triplet excited states were calculated on Gaussian 16 program (Revision A.03) with B3LYP functional and 6-31g(d,p) functional. Spin-orbit coupling (SOC) matrix elements between the singlet excited states and triplet excited states were calculated with spin-orbit mean-field (SOMF) methods on ORCA 5.0.3 program with B3LYP functional and 6-31g(d,p) basis set.

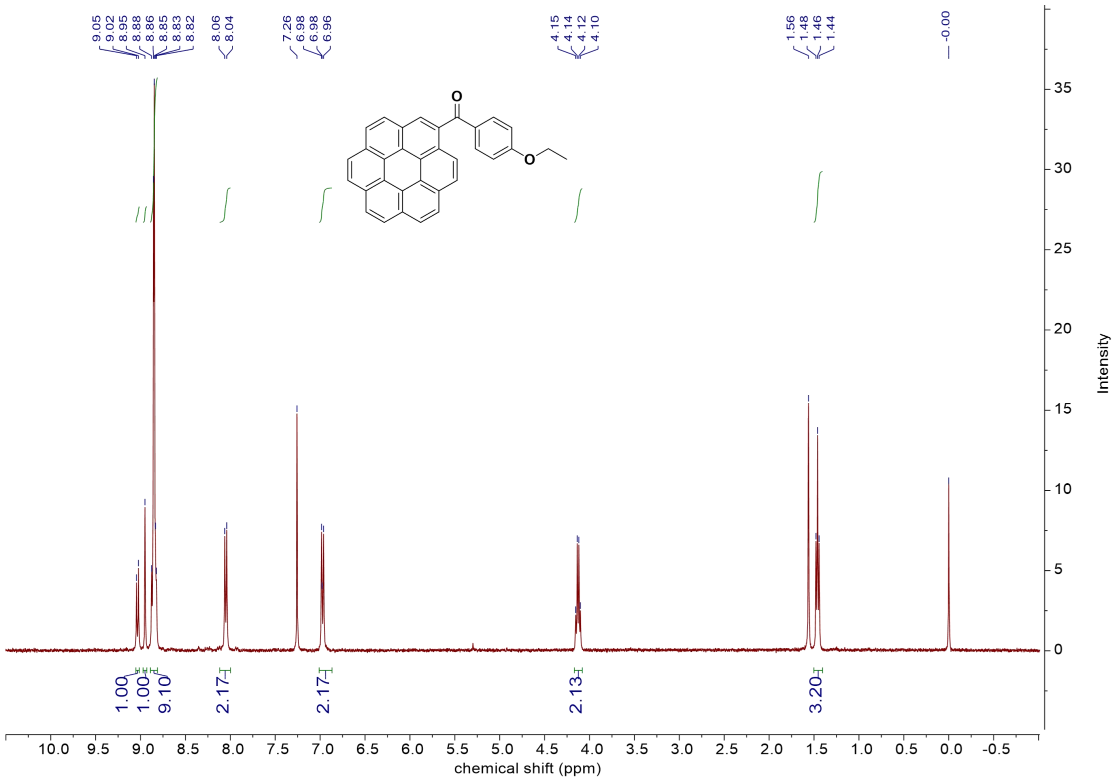

Figure S33. ^{1}H NMR of compound **1** (400 MHz, chloroform-d, 298 K).

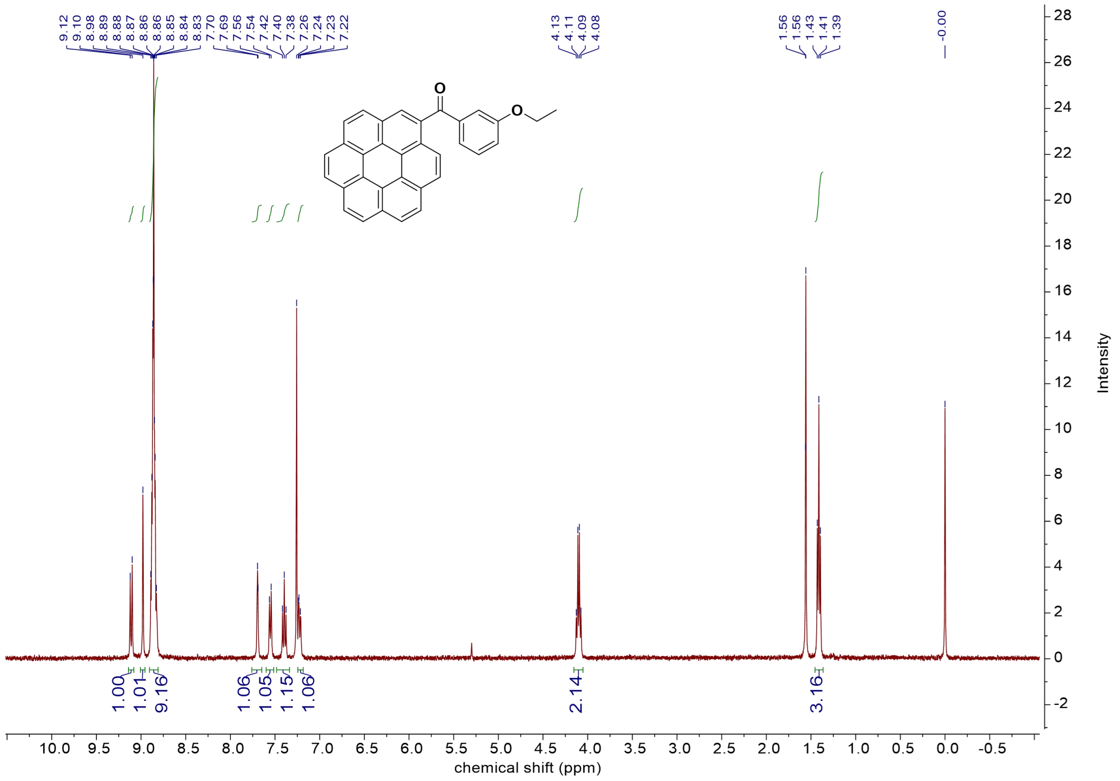

Figure S34. ^{1}H NMR of compound **2** (400 MHz, chloroform-d, 298 K).

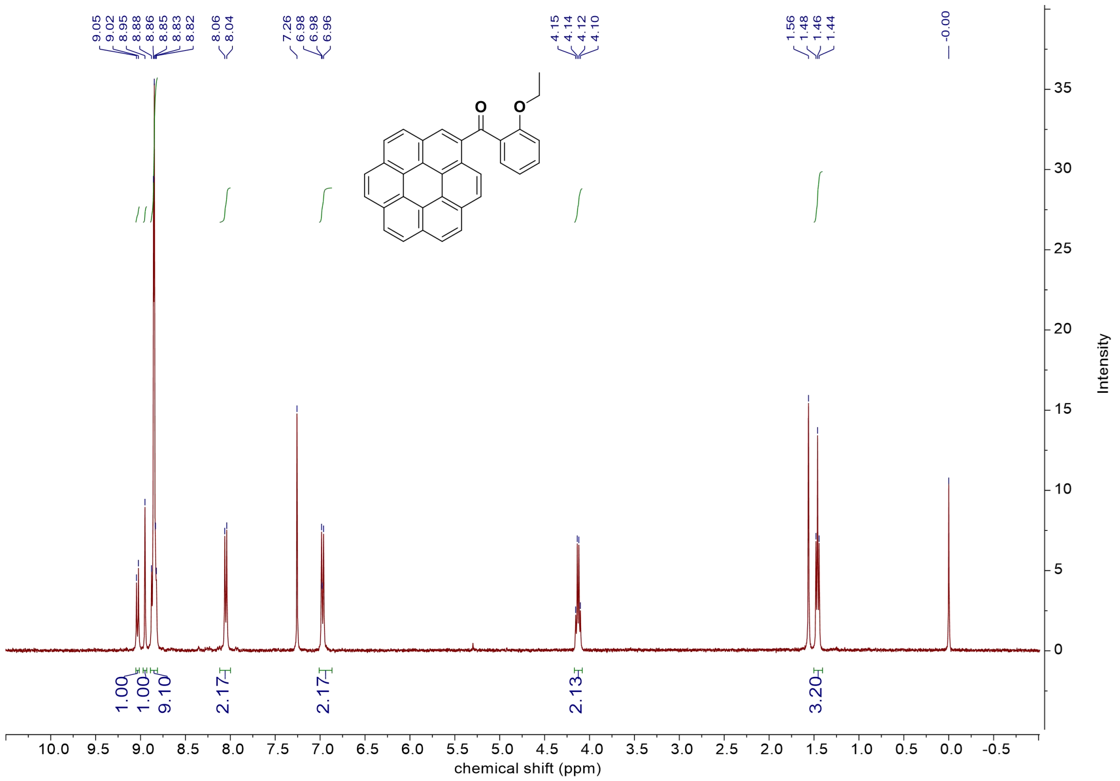

Figure S35. ^{1}H NMR of compound **3** (400 MHz, chloroform-d, 298 K).

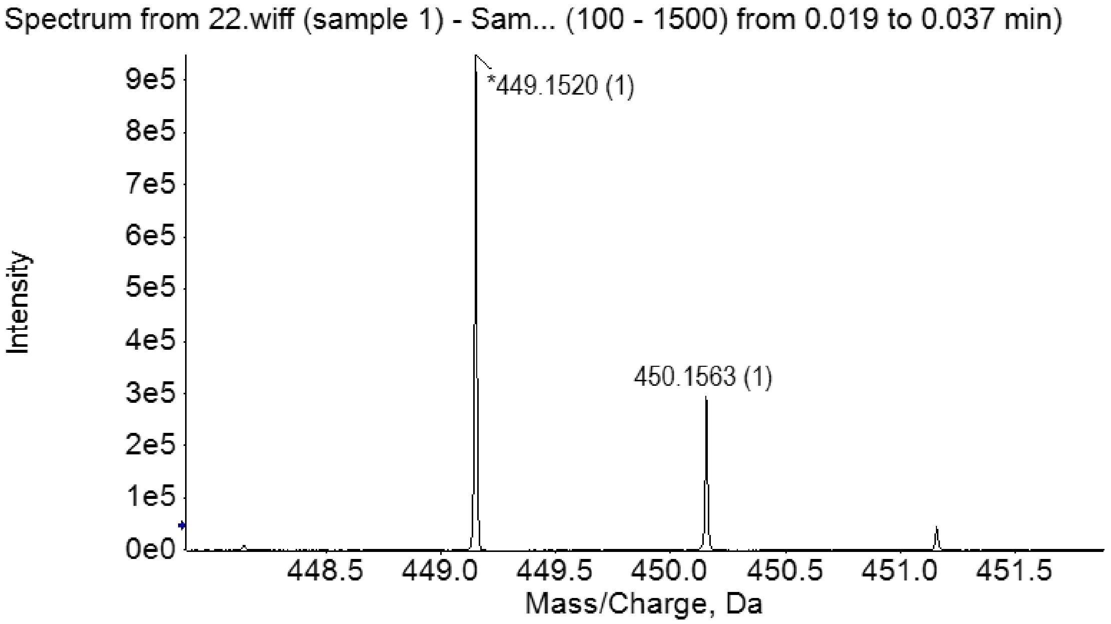

Figure S36. HRMS of compound **1**.

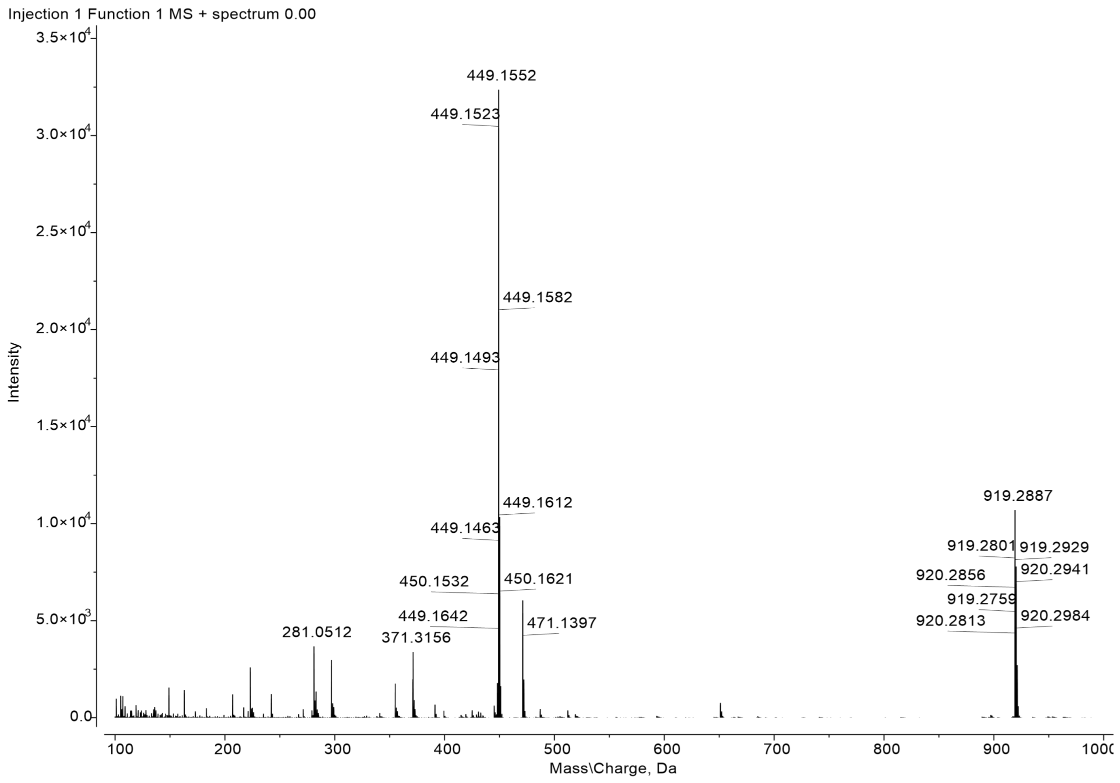

Figure S37. HRMS of compound **2**.

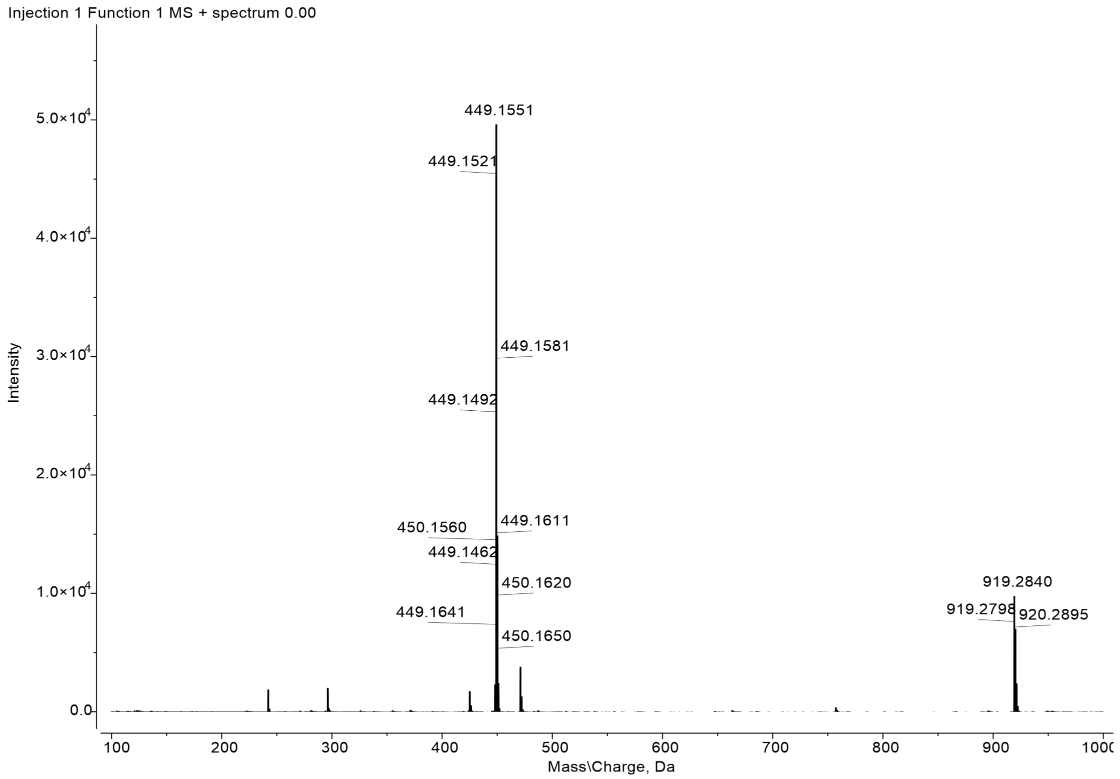

Figure S38. HRMS of compound **3.**

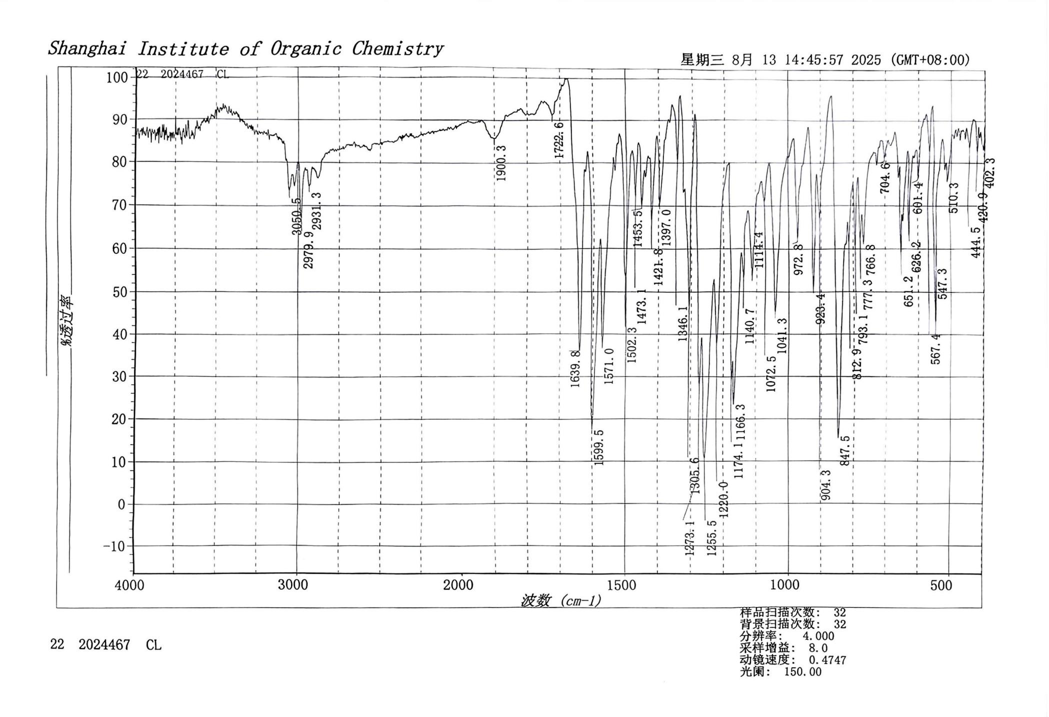

Figure S39. FT-IR spectra of compound **1**.

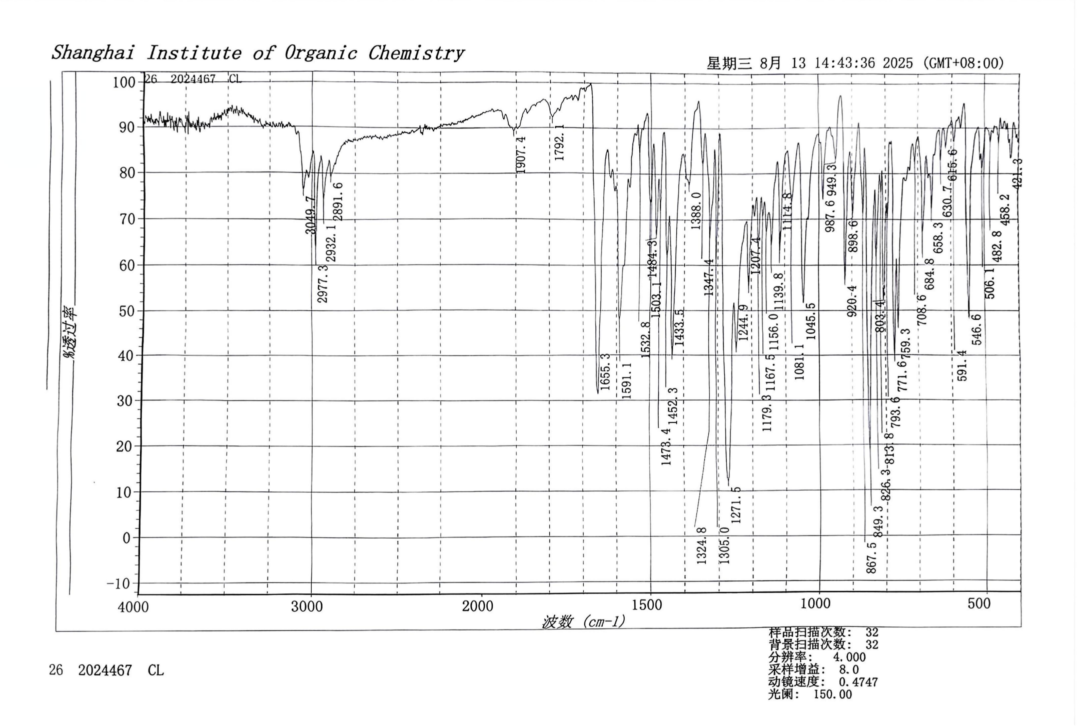

Figure S40. FT-IR spectra of compound **2**.

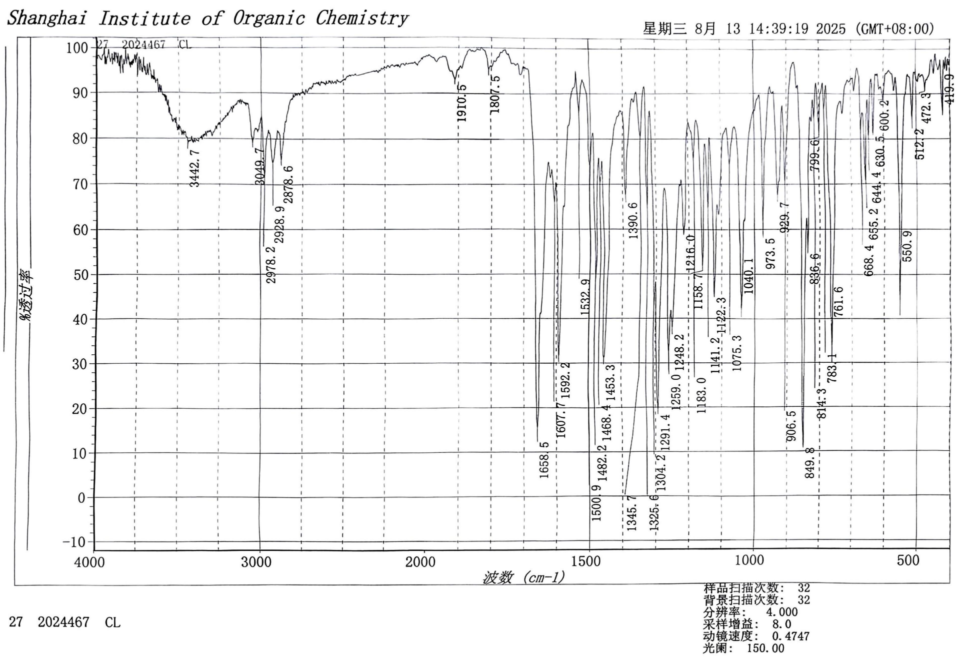

Figure S41. FT-IR spectra of compound **3**.

Table S3. Crystal data and structure refinement for compound **3**.

Identification code	mo_d8v25343_0m	
Empirical formula	C33 H20 O2	
Formula weight	448.49	
Temperature	143 K	
Wavelength	0.71073 Å	
Crystal system	Monoclinic	
Space group	P 1 21/n 1	
Unit cell dimensions	a = 15.4084(9) Å	α= 90°.
	b = 7.9638(5) Å	β= 112.025(2)°.
	c = 18.8192(12) Å	γ = 90°.
Volume	2140.8(2) $Å^3$	
Z	4	
Density (calculated)	1.392 Mg/m^3	
Absorption coefficient	0.085 mm^{-1}	
F(000)	936	
Crystal size	0.17 x 0.17 x 0.05 mm^3	
Theta range for data collection	2.155 to 27.593°.	
Index ranges	-20<=h<=17, -10<=k<=9, -19<=l<=24	
Reflections collected	19126	

Independent reflections	4832 [R(int) = 0.0511]
Completeness to theta = 25.242°	97.6 %
Absorption correction	Semi-empirical from equivalents
Max. and min. transmission	0.7456 and 0.5458
Refinement method	Full-matrix least-squares on F^2
Data / restraints / parameters	4832 / 0 / 317
Goodness-of-fit on F^2	1.053
Final R indices [I>2sigma(I)]	R1 = 0.0698, wR2 = 0.1686
R indices (all data)	R1 = 0.0812, wR2 = 0.1789
Extinction coefficient	n/a
Largest diff. peak and hole	0.779 and -0.303 e.Å^{-3}

Table S4. Atomic coordinates (x 10^4) and equivalent isotropic displacement parameters ($Å^2$x 10^3) for mo_d8v25343_0m. U(eq) is defined as one third of the trace of the orthogonalized U^{ij} tensor.

	x	y	z	U(eq)
O(2)	4347(1)	7862(2)	5670(1)	24(1)
O(1)	5647(1)	4018(2)	6594(1)	31(1)
C(1)	3782(2)	3717(3)	5132(1)	25(1)
C(2)	3141(2)	3040(3)	4458(1)	26(1)
C(3)	2202(2)	2708(3)	4373(1)	24(1)
C(4)	1558(2)	1973(3)	3704(1)	29(1)
C(5)	646(2)	1611(3)	3641(1)	28(1)
C(6)	351(2)	1976(3)	4248(1)	26(1)
C(7)	-563(2)	1596(3)	4199(1)	30(1)
C(8)	-851(2)	1999(3)	4794(1)	30(1)
C(9)	-242(2)	2813(3)	5461(1)	26(1)
C(10)	-523(2)	3289(3)	6058(1)	28(1)
C(11)	71(2)	4139(3)	6695(1)	28(1)
C(12)	994(2)	4547(3)	6769(1)	23(1)
C(13)	1608(2)	5426(3)	7403(1)	24(1)
C(14)	2519(2)	5780(3)	7473(1)	26(1)
C(15)	2849(1)	5261(3)	6898(1)	22(1)
C(16)	3781(1)	5582(2)	6969(1)	20(1)
C(17)	4112(1)	4992(3)	6417(1)	22(1)
C(18)	3503(1)	4165(3)	5744(1)	22(1)
C(19)	2556(1)	3892(3)	5661(1)	22(1)
C(20)	1912(2)	3113(3)	4982(1)	22(1)
C(21)	978(2)	2760(3)	4919(1)	23(1)
C(22)	684(1)	3196(3)	5518(1)	23(1)
C(23)	1297(2)	4043(3)	6171(1)	22(1)
C(24)	2229(2)	4398(2)	6250(1)	21(1)
C(25)	5146(1)	5205(2)	6577(1)	19(1)
C(26)	5543(1)	6932(2)	6772(1)	15(1)
C(27)	6382(1)	7195(2)	7388(1)	17(1)
C(28)	6761(1)	8798(3)	7558(1)	20(1)
C(29)	6289(1)	10141(3)	7105(1)	19(1)
C(30)	5457(1)	9910(2)	6481(1)	18(1)
C(31)	5093(1)	8288(2)	6305(1)	16(1)

C(32)	3775(1)	9148(3)	5187(1)	23(1)
C(33)	3072(2)	8233(3)	4518(1)	29(1)

Table S5. Bond lengths [Å] and angles [°] for compound **3**.

O(2)-C(31)	1.355(2)
O(2)-C(32)	1.432(2)
O(1)-C(25)	1.213(3)
C(1)-H(1)	0.9500
C(1)-C(2)	1.392(3)
C(1)-C(18)	1.416(3)
C(2)-H(2)	0.9500
C(2)-C(3)	1.419(3)
C(3)-C(4)	1.406(3)
C(3)-C(20)	1.414(3)
C(4)-H(4)	0.9500
C(4)-C(5)	1.396(3)
C(5)-H(5)	0.9500
C(5)-C(6)	1.409(3)
C(6)-C(7)	1.409(3)
C(6)-C(21)	1.415(3)
C(7)-H(7)	0.9500
C(7)-C(8)	1.388(4)
C(8)-H(8)	0.9500
C(8)-C(9)	1.411(3)
C(9)-C(10)	1.399(3)
C(9)-C(22)	1.423(3)
C(10)-H(10)	0.9500
C(10)-C(11)	1.382(3)
C(11)-H(11)	0.9500
C(11)-C(12)	1.414(3)
C(12)-C(13)	1.401(3)
C(12)-C(23)	1.429(3)
C(13)-H(13)	0.9500
C(13)-C(14)	1.387(3)
C(14)-H(14)	0.9500
C(14)-C(15)	1.419(3)

C(15)-C(16)	1.415(3)
C(15)-C(24)	1.414(3)
C(16)-H(16)	0.9500
C(16)-C(17)	1.398(3)
C(17)-C(18)	1.425(3)
C(17)-C(25)	1.517(3)
C(18)-C(19)	1.425(3)
C(19)-C(20)	1.432(3)
C(19)-C(24)	1.437(3)
C(20)-C(21)	1.428(3)
C(21)-C(22)	1.406(3)
C(22)-C(23)	1.410(3)
C(23)-C(24)	1.416(3)
C(25)-C(26)	1.494(3)
C(26)-C(27)	1.391(3)
C(26)-C(31)	1.401(3)
C(27)-H(27)	0.9500
C(27)-C(28)	1.390(3)
C(28)-H(28)	0.9500
C(28)-C(29)	1.391(3)
C(29)-H(29)	0.9500
C(29)-C(30)	1.389(3)
C(30)-H(30)	0.9500
C(30)-C(31)	1.398(3)
C(32)-H(32A)	0.9900
C(32)-H(32B)	0.9900
C(32)-C(33)	1.505(3)
C(33)-H(33A)	0.9800
C(33)-H(33B)	0.9800
C(33)-H(33C)	0.9800

C(31)-O(2)-C(32)	119.85(16)
C(2)-C(1)-H(1)	119.6
C(2)-C(1)-C(18)	120.7(2)
C(18)-C(1)-H(1)	119.6
C(1)-C(2)-H(2)	119.4
C(1)-C(2)-C(3)	121.27(19)
C(3)-C(2)-H(2)	119.4

C(4)-C(3)-C(2)	121.6(2)
C(4)-C(3)-C(20)	119.3(2)
C(20)-C(3)-C(2)	119.0(2)
C(3)-C(4)-H(4)	119.6
C(5)-C(4)-C(3)	120.7(2)
C(5)-C(4)-H(4)	119.6
C(4)-C(5)-H(5)	119.7
C(4)-C(5)-C(6)	120.7(2)
C(6)-C(5)-H(5)	119.7
C(5)-C(6)-C(7)	121.4(2)
C(5)-C(6)-C(21)	119.6(2)
C(7)-C(6)-C(21)	118.9(2)
C(6)-C(7)-H(7)	119.6
C(8)-C(7)-C(6)	120.8(2)
C(8)-C(7)-H(7)	119.6
C(7)-C(8)-H(8)	119.5
C(7)-C(8)-C(9)	121.1(2)
C(9)-C(8)-H(8)	119.5
C(8)-C(9)-C(22)	118.6(2)
C(10)-C(9)-C(8)	122.3(2)
C(10)-C(9)-C(22)	119.0(2)
C(9)-C(10)-H(10)	119.1
C(11)-C(10)-C(9)	121.7(2)
C(11)-C(10)-H(10)	119.1
C(10)-C(11)-H(11)	119.6
C(10)-C(11)-C(12)	120.8(2)
C(12)-C(11)-H(11)	119.6
C(11)-C(12)-C(23)	118.1(2)
C(13)-C(12)-C(11)	122.2(2)
C(13)-C(12)-C(23)	119.7(2)
C(12)-C(13)-H(13)	119.4
C(14)-C(13)-C(12)	121.26(19)
C(14)-C(13)-H(13)	119.4
C(13)-C(14)-H(14)	119.8
C(13)-C(14)-C(15)	120.5(2)
C(15)-C(14)-H(14)	119.8
C(16)-C(15)-C(14)	121.14(19)
C(24)-C(15)-C(14)	118.73(19)

C(24)-C(15)-C(16)	120.13(19)
C(15)-C(16)-H(16)	119.7
C(17)-C(16)-C(15)	120.62(19)
C(17)-C(16)-H(16)	119.7
C(16)-C(17)-C(18)	120.99(19)
C(16)-C(17)-C(25)	117.67(18)
C(18)-C(17)-C(25)	121.31(19)
C(1)-C(18)-C(17)	122.76(19)
C(1)-C(18)-C(19)	118.9(2)
C(17)-C(18)-C(19)	118.23(19)
C(18)-C(19)-C(20)	120.16(19)
C(18)-C(19)-C(24)	120.94(19)
C(20)-C(19)-C(24)	118.90(18)
C(3)-C(20)-C(19)	119.8(2)
C(3)-C(20)-C(21)	120.2(2)
C(21)-C(20)-C(19)	119.98(18)
C(6)-C(21)-C(20)	119.4(2)
C(22)-C(21)-C(6)	120.5(2)
C(22)-C(21)-C(20)	120.14(19)
C(21)-C(22)-C(9)	120.1(2)
C(21)-C(22)-C(23)	120.38(19)
C(23)-C(22)-C(9)	119.5(2)
C(22)-C(23)-C(12)	120.81(19)
C(22)-C(23)-C(24)	120.52(19)
C(24)-C(23)-C(12)	118.7(2)
C(15)-C(24)-C(19)	118.90(19)
C(15)-C(24)-C(23)	121.15(19)
C(23)-C(24)-C(19)	119.95(18)
O(1)-C(25)-C(17)	121.94(18)
O(1)-C(25)-C(26)	120.88(18)
C(26)-C(25)-C(17)	117.01(17)
C(27)-C(26)-C(25)	120.72(17)
C(27)-C(26)-C(31)	119.54(17)
C(31)-C(26)-C(25)	119.64(17)
C(26)-C(27)-H(27)	119.7
C(28)-C(27)-C(26)	120.60(18)
C(28)-C(27)-H(27)	119.7
C(27)-C(28)-H(28)	120.4

C(27)-C(28)-C(29)	119.19(18)
C(29)-C(28)-H(28)	120.4
C(28)-C(29)-H(29)	119.3
C(30)-C(29)-C(28)	121.39(18)
C(30)-C(29)-H(29)	119.3
C(29)-C(30)-H(30)	120.5
C(29)-C(30)-C(31)	118.92(18)
C(31)-C(30)-H(30)	120.5
O(2)-C(31)-C(26)	114.66(17)
O(2)-C(31)-C(30)	124.91(17)
C(30)-C(31)-C(26)	120.28(17)
O(2)-C(32)-H(32A)	110.7
O(2)-C(32)-H(32B)	110.7
O(2)-C(32)-C(33)	105.27(17)
H(32A)-C(32)-H(32B)	108.8
C(33)-C(32)-H(32A)	110.7
C(33)-C(32)-H(32B)	110.7
C(32)-C(33)-H(33A)	109.5
C(32)-C(33)-H(33B)	109.5
C(32)-C(33)-H(33C)	109.5
H(33A)-C(33)-H(33B)	109.5
H(33A)-C(33)-H(33C)	109.5
H(33B)-C(33)-H(33C)	109.5

Symmetry transformations used to generate equivalent atoms:

Table S6. Anisotropic displacement parameters ($Å^2 x 10^3$) for compound **3**. The anisotropic displacement factor exponent takes the form: $-2\pi^2 [h^2 a^{*2}U^{11} + ... + 2 h k a^* b^* U^{12}]$

	U^{11}	U^{22}	U^{33}	U^{23}	U^{13}	U^{12}
O(2)	23(1)	16(1)	22(1)	4(1)	-4(1)	-2(1)
O(1)	26(1)	16(1)	50(1)	0(1)	15(1)	3(1)
C(1)	26(1)	19(1)	36(1)	4(1)	20(1)	1(1)
C(2)	34(1)	24(1)	29(1)	5(1)	21(1)	4(1)
C(3)	31(1)	19(1)	23(1)	3(1)	10(1)	2(1)
C(4)	44(1)	23(1)	21(1)	1(1)	14(1)	3(1)
C(5)	37(1)	23(1)	17(1)	-3(1)	0(1)	-3(1)
C(6)	26(1)	21(1)	25(1)	3(1)	3(1)	-2(1)
C(7)	27(1)	25(1)	27(1)	0(1)	-3(1)	-5(1)
C(8)	18(1)	31(1)	38(1)	9(1)	6(1)	-4(1)
C(9)	22(1)	23(1)	29(1)	7(1)	7(1)	-2(1)
C(10)	16(1)	27(1)	39(1)	8(1)	11(1)	-1(1)
C(11)	29(1)	30(1)	30(1)	7(1)	18(1)	6(1)
C(12)	25(1)	21(1)	24(1)	3(1)	10(1)	2(1)
C(13)	31(1)	24(1)	22(1)	1(1)	14(1)	5(1)
C(14)	30(1)	24(1)	22(1)	-5(1)	7(1)	-3(1)
C(15)	22(1)	19(1)	23(1)	1(1)	7(1)	0(1)
C(16)	18(1)	15(1)	22(1)	2(1)	2(1)	-2(1)
C(17)	20(1)	15(1)	29(1)	5(1)	7(1)	-1(1)
C(18)	23(1)	17(1)	27(1)	3(1)	10(1)	0(1)
C(19)	18(1)	16(1)	31(1)	10(1)	10(1)	1(1)
C(20)	35(1)	15(1)	22(1)	6(1)	16(1)	6(1)
C(21)	25(1)	15(1)	22(1)	5(1)	0(1)	-2(1)
C(22)	20(1)	18(1)	31(1)	10(1)	8(1)	2(1)
C(23)	28(1)	15(1)	26(1)	7(1)	14(1)	5(1)
C(24)	27(1)	13(1)	18(1)	3(1)	4(1)	-1(1)
C(25)	19(1)	15(1)	20(1)	3(1)	5(1)	-1(1)
C(26)	13(1)	14(1)	18(1)	1(1)	7(1)	-1(1)
C(27)	13(1)	19(1)	20(1)	4(1)	6(1)	2(1)
C(28)	14(1)	24(1)	19(1)	-2(1)	4(1)	-3(1)
C(29)	19(1)	17(1)	22(1)	-3(1)	9(1)	-4(1)
C(30)	19(1)	14(1)	20(1)	2(1)	7(1)	0(1)
C(31)	15(1)	17(1)	17(1)	2(1)	5(1)	0(1)

C(32)	20(1)	20(1)	23(1)	5(1)	1(1)	3(1)
C(33)	22(1)	30(1)	25(1)	5(1)	-3(1)	-1(1)

Table S7. Hydrogen coordinates (x 10^4) and isotropic displacement parameters ($Å^2$x 10^{3}) for compound **3**.

	x	y	z	U(eq)
H(1)	4413	3881	5183	30
H(2)	3335	2796	4046	31
H(4)	1746	1720	3289	35
H(5)	219	1115	3185	34
H(7)	-987	1057	3754	36
H(8)	-1468	1722	4752	36
H(10)	-1138	3020	6024	33
H(11)	-144	4453	7088	34
H(13)	1398	5787	7792	29
H(14)	2924	6373	7910	31
H(16)	4185	6205	7395	24
H(27)	6700	6271	7694	21
H(28)	7334	8974	7978	24
H(29)	6542	11240	7225	23
H(30)	5140	10840	6178	21
H(32A)	4158	9919	5011	27
H(32B)	3456	9806	5464	27
H(33A)	2612	9035	4191	43
H(33B)	2751	7384	4707	43
H(33C)	3395	7685	4220	43

Table S8. Torsion angles [°] for compound **3**.

O(1)-C(25)-C(26)-C(27)	-41.7(3)
O(1)-C(25)-C(26)-C(31)	134.6(2)
C(1)-C(2)-C(3)-C(4)	-177.9(2)
C(1)-C(2)-C(3)-C(20)	1.5(3)
C(1)-C(18)-C(19)-C(20)	2.7(3)
C(1)-C(18)-C(19)-C(24)	-177.04(18)
C(2)-C(1)-C(18)-C(17)	-174.5(2)
C(2)-C(1)-C(18)-C(19)	0.7(3)
C(2)-C(3)-C(4)-C(5)	178.1(2)
C(2)-C(3)-C(20)-C(19)	1.9(3)
C(2)-C(3)-C(20)-C(21)	-178.71(19)
C(3)-C(4)-C(5)-C(6)	0.0(3)
C(3)-C(20)-C(21)-C(6)	1.2(3)
C(3)-C(20)-C(21)-C(22)	-178.59(18)
C(4)-C(3)-C(20)-C(19)	-178.73(19)
C(4)-C(3)-C(20)-C(21)	0.6(3)
C(4)-C(5)-C(6)-C(7)	-179.0(2)
C(4)-C(5)-C(6)-C(21)	1.8(3)
C(5)-C(6)-C(7)-C(8)	-178.4(2)
C(5)-C(6)-C(21)-C(20)	-2.4(3)
C(5)-C(6)-C(21)-C(22)	177.4(2)
C(6)-C(7)-C(8)-C(9)	0.6(3)
C(6)-C(21)-C(22)-C(9)	1.6(3)
C(6)-C(21)-C(22)-C(23)	-177.24(19)
C(7)-C(6)-C(21)-C(20)	178.44(19)
C(7)-C(6)-C(21)-C(22)	-1.8(3)
C(7)-C(8)-C(9)-C(10)	177.7(2)
C(7)-C(8)-C(9)-C(22)	-0.9(3)
C(8)-C(9)-C(10)-C(11)	-177.7(2)
C(8)-C(9)-C(22)-C(21)	-0.2(3)
C(8)-C(9)-C(22)-C(23)	178.59(19)
C(9)-C(10)-C(11)-C(12)	-0.6(3)
C(9)-C(22)-C(23)-C(12)	-1.2(3)
C(9)-C(22)-C(23)-C(24)	178.31(19)
C(10)-C(9)-C(22)-C(21)	-178.80(19)
C(10)-C(9)-C(22)-C(23)	0.0(3)

C(10)-C(11)-C(12)-C(13)	179.2(2)
C(10)-C(11)-C(12)-C(23)	-0.5(3)
C(11)-C(12)-C(13)-C(14)	178.5(2)
C(11)-C(12)-C(23)-C(22)	1.4(3)
C(11)-C(12)-C(23)-C(24)	-178.06(19)
C(12)-C(13)-C(14)-C(15)	0.3(3)
C(12)-C(23)-C(24)-C(15)	-1.2(3)
C(12)-C(23)-C(24)-C(19)	179.42(18)
C(13)-C(12)-C(23)-C(22)	-178.31(19)
C(13)-C(12)-C(23)-C(24)	2.2(3)
C(13)-C(14)-C(15)-C(16)	-178.7(2)
C(13)-C(14)-C(15)-C(24)	0.8(3)
C(14)-C(15)-C(16)-C(17)	176.78(19)
C(14)-C(15)-C(24)-C(19)	179.09(19)
C(14)-C(15)-C(24)-C(23)	-0.3(3)
C(15)-C(16)-C(17)-C(18)	4.8(3)
C(15)-C(16)-C(17)-C(25)	-173.00(18)
C(16)-C(15)-C(24)-C(19)	-1.4(3)
C(16)-C(15)-C(24)-C(23)	179.19(19)
C(16)-C(17)-C(18)-C(1)	172.68(19)
C(16)-C(17)-C(18)-C(19)	-2.6(3)
C(16)-C(17)-C(25)-O(1)	122.4(2)
C(16)-C(17)-C(25)-C(26)	-53.1(2)
C(17)-C(18)-C(19)-C(20)	178.10(18)
C(17)-C(18)-C(19)-C(24)	-1.6(3)
C(17)-C(25)-C(26)-C(27)	133.76(19)
C(17)-C(25)-C(26)-C(31)	-49.9(2)
C(18)-C(1)-C(2)-C(3)	-2.8(3)
C(18)-C(17)-C(25)-O(1)	-55.4(3)
C(18)-C(17)-C(25)-C(26)	129.2(2)
C(18)-C(19)-C(20)-C(3)	-4.0(3)
C(18)-C(19)-C(20)-C(21)	176.65(18)
C(18)-C(19)-C(24)-C(15)	3.5(3)
C(18)-C(19)-C(24)-C(23)	-177.01(18)
C(19)-C(20)-C(21)-C(6)	-179.45(18)
C(19)-C(20)-C(21)-C(22)	0.8(3)
C(20)-C(3)-C(4)-C(5)	-1.2(3)
C(20)-C(19)-C(24)-C(15)	-176.15(18)

C(20)-C(19)-C(24)-C(23)	3.3(3)
C(20)-C(21)-C(22)-C(9)	-178.69(18)
C(20)-C(21)-C(22)-C(23)	2.5(3)
C(21)-C(6)-C(7)-C(8)	0.7(3)
C(21)-C(22)-C(23)-C(12)	177.65(19)
C(21)-C(22)-C(23)-C(24)	-2.9(3)
C(22)-C(9)-C(10)-C(11)	0.9(3)
C(22)-C(23)-C(24)-C(15)	179.37(18)
C(22)-C(23)-C(24)-C(19)	-0.1(3)
C(23)-C(12)-C(13)-C(14)	-1.8(3)
C(24)-C(15)-C(16)-C(17)	-2.7(3)
C(24)-C(19)-C(20)-C(3)	175.73(18)
C(24)-C(19)-C(20)-C(21)	-3.6(3)
C(25)-C(17)-C(18)-C(1)	-9.6(3)
C(25)-C(17)-C(18)-C(19)	175.13(18)
C(25)-C(26)-C(27)-C(28)	178.53(18)
C(25)-C(26)-C(31)-O(2)	-4.2(3)
C(25)-C(26)-C(31)-C(30)	-179.92(17)
C(26)-C(27)-C(28)-C(29)	0.0(3)
C(27)-C(26)-C(31)-O(2)	172.13(17)
C(27)-C(26)-C(31)-C(30)	-3.6(3)
C(27)-C(28)-C(29)-C(30)	-0.9(3)
C(28)-C(29)-C(30)-C(31)	-0.4(3)
C(29)-C(30)-C(31)-O(2)	-172.59(18)
C(29)-C(30)-C(31)-C(26)	2.7(3)
C(31)-O(2)-C(32)-C(33)	175.82(18)
C(31)-C(26)-C(27)-C(28)	2.2(3)
C(32)-O(2)-C(31)-C(26)	173.92(17)
C(32)-O(2)-C(31)-C(30)	-10.6(3)

Symmetry transformations used to generate equivalent atoms:

Supplementary References

1. M. J. Frisch *et al.*, Gaussian 16, Rev. A.03. *Gaussian, Inc., Wallingford, CT* **2016**.
2. F. Neese, F. Wennmohs, U. Becker and C. Riplinger, *J. Chem. Phys.*, **2020**, *152*, 224108.
3. F. Neese, *WIREs Comput. Mol. Sci.*, **2022**, *12*, e1606.
4. W. Humphrey, A. Dalke and K. Schulten, *J. Mol. Graphics*, **1996**, *14*, 33-38.
5. T. Lu and F. Chen, *J. Comput. Chem.*, **2012**, *33*, 580-592.
6. Z. Liu, T. Lu and Q. Chen, *Carbon*, **2020**, *165*, 461-467.
7. Cerezo J. and Santoro F., *J. Comput. Chem.*, **2023**, *44*, 626-643.
